# Integrated transcriptomics and epigenomics reveal chamber-specific and species-specific characteristics of human and mouse hearts

**DOI:** 10.1371/journal.pbio.3001229

**Published:** 2021-05-18

**Authors:** Junpeng Gao, Yuxuan Zheng, Lin Li, Minjie Lu, Xiangjian Chen, Yu Wang, Yanna Li, Xiaomeng Liu, Yun Gao, Yunuo Mao, Peng Zhao, Jinan Zhang, Fuchou Tang, Lei Song, Lu Wen, Jizheng Wang

**Affiliations:** 1 Beijing Advanced Innovation Center for Genomics, School of Life Sciences, Peking University, Beijing, China; 2 Biomedical Pioneering Innovation Center, Ministry of Education Key Laboratory of Cell Proliferation and Differentiation, Beijing, China; 3 Peking-Tsinghua Center for Life Sciences, Academy for Advanced Interdisciplinary Studies, Peking University, Beijing, China; 4 Guangdong Provincial Key Laboratory of Proteomics, Department of Pathophysiology, School of Basic Medical Sciences, Southern Medical University, Guangzhou, China; 5 State Key Laboratory of Cardiovascular Disease, Fuwai Hospital, National Center for Cardiovascular Diseases, Chinese Academy of Medical Sciences and Peking Union Medical College, Beijing, China; 6 Department of Cardiology, First Affiliated Hospital of Nanjing Medical University, Nanjing, China; 7 Department of Ultrasound, Xiangyang No.1 People’s Hospital, Hubei University of Medicine, Xiangyang, China; 8 Department of Obstetric and Gynecology, Beijing Anzhen Hospital, Capital Medical University, Beijing, China; 9 Sichuan Cancer Hospital & Institute, Sichuan Cancer Center, School of Medicine, University of Electronic Science and Technology of China, Radiation Oncology Key Laboratory Of Sichuan Province, Chengdu, China; 10 Department of Pathology, The Affiliated Hospital of Qingdao University, Qingdao, China; 11 Clinical Research Center for Cardiovascular Diseases, Fuwai Hospital, National Center for Cardiovascular Diseases, Chinese Academy of Medical Sciences and Peking Union Medical College, Beijing, China; University of Pittsburgh, UNITED STATES

## Abstract

DNA methylation, chromatin accessibility, and gene expression represent different levels information in biological process, but a comprehensive multiomics analysis of the mammalian heart is lacking. Here, we applied nucleosome occupancy and methylome sequencing, which detected DNA methylation and chromatin accessibility simultaneously, as well as RNA-seq, for multiomics analysis of the 4 chambers of adult and fetal human hearts, and adult mouse hearts. Our results showed conserved region-specific patterns in the mammalian heart at transcriptome and DNA methylation level. Adult and fetal human hearts showed distinct features in DNA methylome, chromatin accessibility, and transcriptome. Novel long noncoding RNAs were identified in the human heart, and the gene expression profiles of major cardiovascular diseases associated genes were displayed. Furthermore, cross-species comparisons revealed human-specific and mouse-specific differentially expressed genes between the atria and ventricles. We also reported the relationship among multiomics and found there was a bell-shaped relationship between gene-body methylation and expression in the human heart. In general, our study provided comprehensive spatiotemporal and evolutionary insights into the regulation of gene expression in the heart.

## Abstract 2

Multi-omic analyses of the four chambers of the human and mouse heart, including transcriptome, DNA methylation and chromatin accessibility, reveals characteristic patterns of gene regulation at the level of heart regions.

## Introduction

The heart is the first organ formed during mammalian embryogenesis [1] and is composed of 4 chambers: the left atrium (LA), the right atrium (RA), the left ventricle (LV), and the right ventricle (RV) [2]. The atria and the ventricles play different roles, and all 4 chambers exhibit distinct properties with respect to structure, biochemistry, and electrochemistry [3,4]. With the rapid development of high-throughput sequencing technologies, genomic studies have emerged to reveal the molecular characteristics of the mammalian heart [5–13]. For example, region-resolved transcriptional and quantitative proteomic maps of the human heart have been reported in recent years [14–19]. However, all these studies have focused on individual omics, especially transcriptomics. DNA methylation and other epigenetic aspects, which are essential for cardiogenesis and cardiovascular disease, have not been sufficiently investigated. In addition to this, a few studies seek to resolve cardiac physiology and pathology at the multiomics level [20,21]. But a comprehensive multiomics analysis comparing DNA methylation, chromatin accessibility, and transcriptome at one time has not been reported for 4 chambers of mammalian hearts.

Previous studies have revealed the fetal origins of cardiovascular disease [22,23]. Heart development is a complex process that is controlled by interacting molecular pathways [24]. The formation of all 4 chambers is completed before the ninth week of human embryogenesis [25]. Current research has mainly concentrated on the process of embryonic heart development before birth or on regional differences in the adult human heart [26]. Because of their similarity to humans in physiology and pathology, mice are the most commonly used model organisms in human biology and disease research [27]. However, the chamber-specific characteristics and the species differences between human and mouse hearts have not yet been comprehensively investigated, although these data could support decision-making in drug discovery.

In the past few years, a large number of techniques have arisen to map the DNA methylome and chromatin accessibility at the whole-genome scale. For example, whole genome bisulfite sequencing (WGBS), post-bisulfite adaptor tagging (PBAT), and reduced representation bisulfite sequencing (RRBS) have been widely used to investigate the state of DNA methylation at single-base resolution [28–30]. DNase sequencing (DNase-Seq), micrococcal nuclease sequencing (MNase-seq), and assay for transposase-accessible chromatin using sequencing (ATAC-seq) are several ways to identify chromatin organization [31–33]. Here, we performed multiomics analyses of the 4 chambers of both human and mouse hearts using nucleosome occupancy and methylome sequencing (NOMe-seq), which simultaneously detects DNA methylation and chromatin accessibility [34], as well as RNA-seq. Our results revealed chamber-specific and species-specific characteristics of gene expression, DNA methylation, and chromatin accessibility of human and mouse hearts. The resulting multiomics map of healthy human and mouse hearts can be used as a reference to identify novel biomarkers or drug targets when compared with malfunctioning hearts.

## Results

### Multiomics analyses of human and mouse hearts

To obtain an integrated multiomics map of mammalian hearts, we collected 11 adult human hearts from healthy donors who contributed for other reasons instead of cardiovascular disease and 3 fetal human hearts from aborted fetuses. We also harvested hearts from 3 groups of male C57BL/6 mice (10 weeks old) with 5 mice in each group. Four chambers, i.e., the LA, RA, LV, and RV, were separately analyzed for each sample.

**Fig 1:**
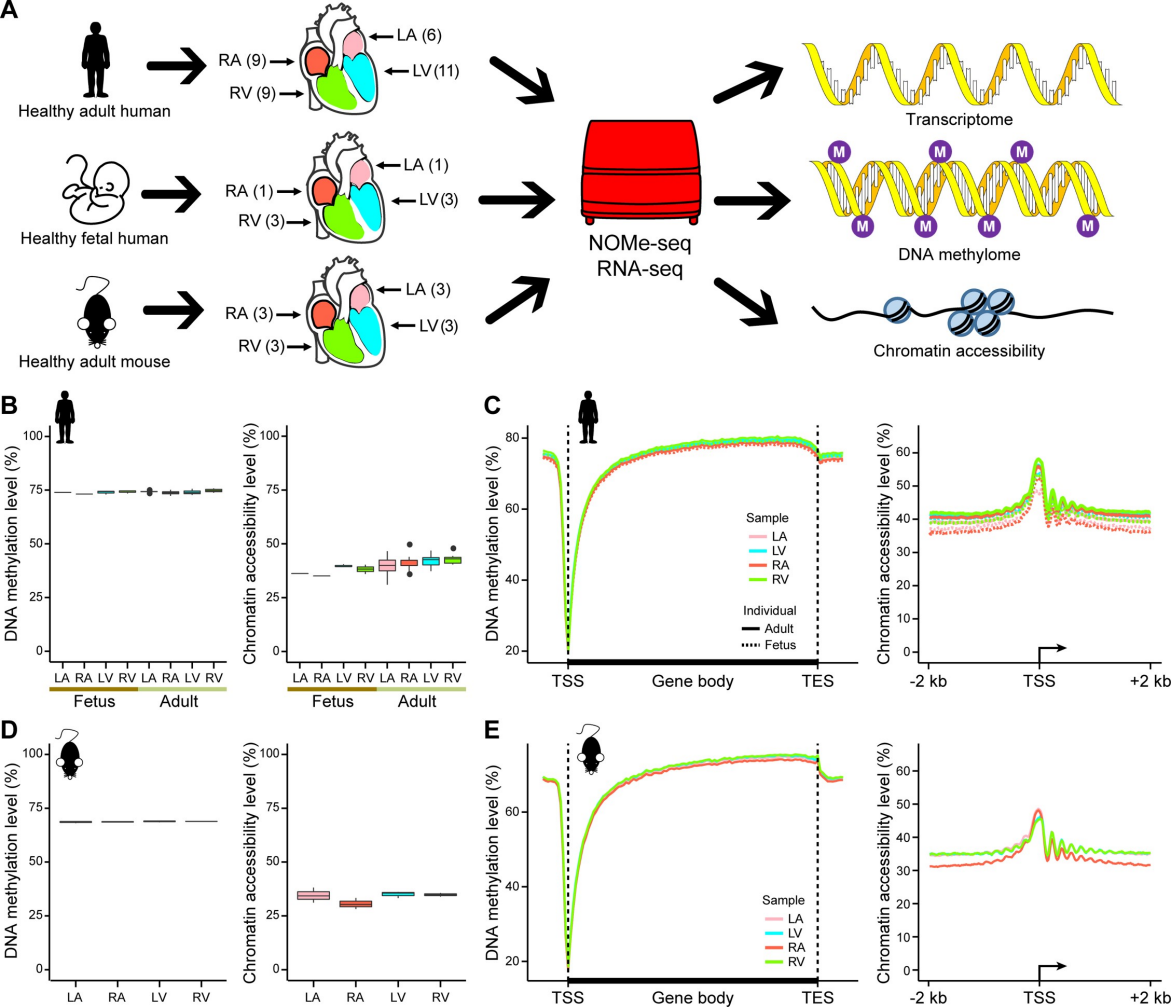
Global features of DNA methylation and chromatin accessibility in human and mouse hearts. (A) Experimental design, including heart sources and schematic depiction of the multiomics analysis. Numbers in brackets refer to the numbers of hearts used in the study. (B) Boxplots showing the endogenous DNA methylation level (left) and chromatin accessibility levels (right) in the human heart. The endogenous DNA methylation level was calculated using the DNA methylation level of WCG sites (ACG and TCG trinucleotides), and the chromatin accessibility level was calculated using the DNA methylation level of GCH sites (GCA, GCT, and GCC trinucleotides). (C) Line plots showing the average endogenous DNA methylation level around the gene body ± 5 kb region (left) and the average chromatin accessibility levels around the TSS ± 2 kb region (right) in the human heart. Different colors indicate different anatomic regions, and the solid line and dashed line indicate adult samples and fetal samples, respectively. (D) Boxplots showing the endogenous DNA methylation level (left) and the chromatin accessibility levels (right) in the mouse heart. (E) Line plots showing the average endogenous DNA methylation level around the gene body ± 5 kb regions (left) and the average chromatin accessibility levels around the TSS ± 2 kb regions (right) in the mouse heart. Different colors indicate different anatomic regions. The raw data for B–E can be found in S1 Data. LA, left atrium; LV, left ventricle; NOMe-seq, nucleosome occupancy and methylome sequencing; RA, right atrium; RV, right ventricle; TES, transcription end site; TSS, transcription start site.

We performed a modified version of NOMe-seq for the heart samples with 2 replicates for each sample. Ribosomal RNA-depleted RNA-seq was also applied for profiling the transcriptome (Fig 1A, see Methods). We sequenced an average of 34.26 Gb raw data for each NOMe-seq library and 8.48 Gb raw data for each RNA-seq library (S1 Table). In total, 4.14 Tb sequencing data were obtained. The results of the technical replicates were highly reproducible, as shown by the DNA methylation patterns around the gene-body region and the chromatin accessibility patterns around the transcription start site (TSS) (S1A and S1B Fig). In human and mouse samples, the median efficiencies of GpC methyltransferase and bisulfite conversion were 92.65% and 95.93%, respectively, as assessed by spiked lambda DNA. These results together indicated that we successfully applied NOMe-seq to the heart tissues, and the data were reliable for subsequent analysis.

First, the total levels of DNA methylation and chromatin accessibility were compared among the 4 chambers of human and mouse hearts. The DNA methylation and chromatin accessibility were calculated using the methylation levels of cytosine in the WCG (ACG/TCG) and GCH (GCA/GCT/GCC) sites, respectively. The results showed that the total DNA methylation levels were comparable among the different chambers of adult and fetal human hearts and adult mouse hearts (Fig 1B–1E, S1C and S1D Fig). The average methylation levels were 74% for humans and 69% for mice, consistent with previous reports [35,36]. The total chromatin accessibility levels were also largely similar among the different chambers of human and mouse hearts (Fig 1E, S1C and S1D Fig). In addition, both the DNA methylation levels and the chromatin accessibility levels were similar between fetal and adult human hearts (Fig 1B and 1C).

### Comparative analysis of the four chambers of the adult human heart

**Fig 2:**
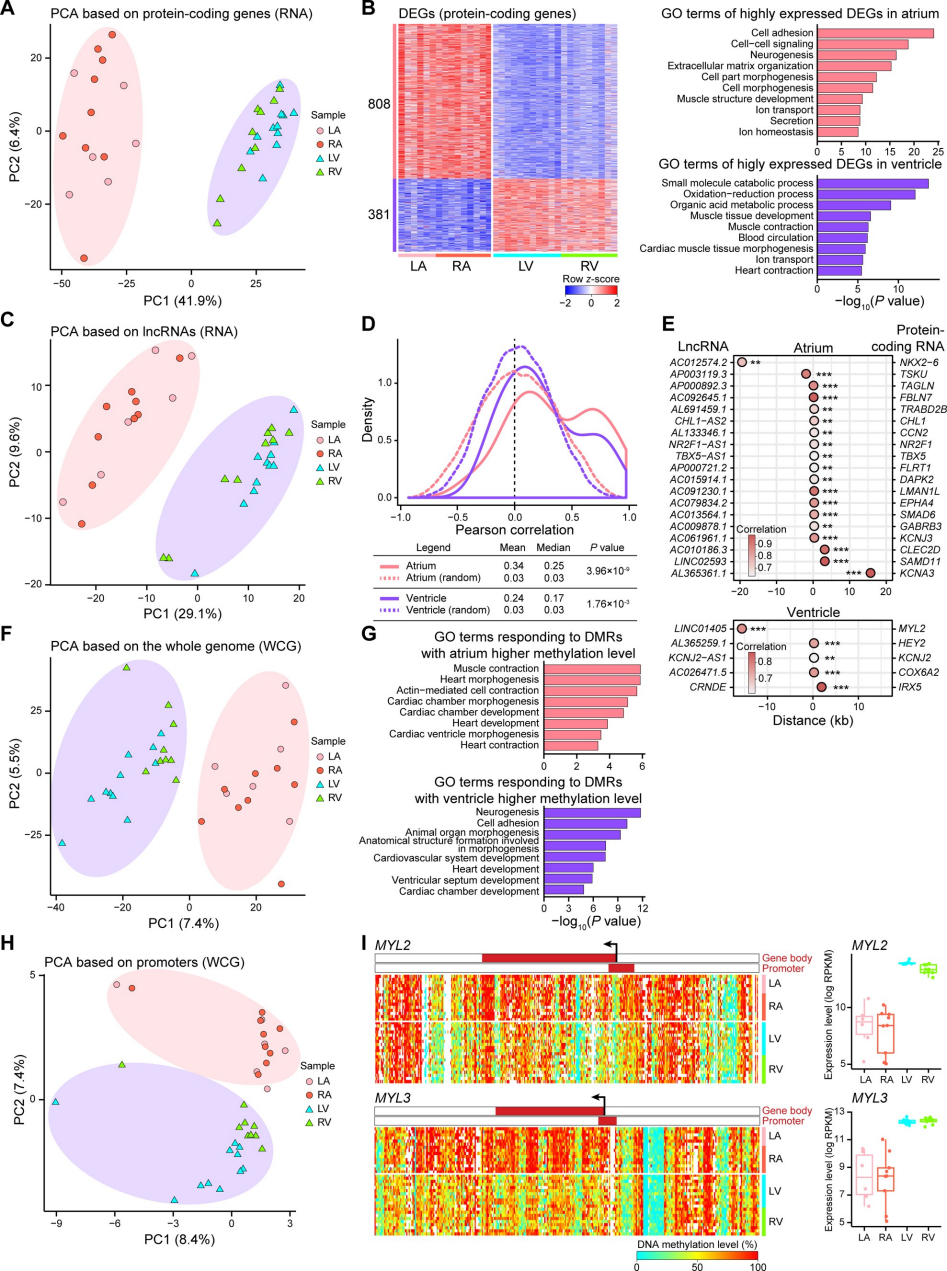
Regional differences in transcriptome and endogenous DNA methylation profiles in the adult human heart. (A) PCA plot showing the transcriptome pattern of protein-coding genes in the adult human heart. Circles indicate atrium samples, and triangles indicate ventricle samples. The variation values of PC1 and PC2 were 41.9% and 6.4%, respectively. (B) Heatmap showing row *z*-score scaled gene expression levels of DEGs (protein-coding genes) between atria and ventricles (left), and corresponding GO terms are shown (right). The number of DEGs is reported on the left. (C) PCA plot showing the transcriptome pattern of lncRNAs in the adult human heart. Circles indicate atrium samples, and triangles indicate ventricle samples. The variation values of PC1 and PC2 were 29.1% and 9.6%, respectively. (D) Line plot showing the density distribution of the Pearson correlation coefficient between the paired protein-coding genes and lncRNAs (top). Solid lines indicate the real distribution, dashed lines indicate the simulated distribution, and pink lines and purple lines correspond to the atria and ventricles, respectively. The mean and median Pearson correlation coefficients are reported, and two-tailed Student *t* test *P* values between the real and simulated distributions are indicated (bottom). (E) Dot plots showing gene pairs between lncRNAs (left) and protein-coding genes (right) in *cis*-regulation analysis in the atria (top) and the ventricles (bottom). The x-axis indicates the linear distance between the lncRNA and the protein-coding genes. Dot colors indicate the Pearson correlation coefficient, and stars indicate the correlation significance. ** *P* value ≤ 0.01 and *** *P* value ≤ 0.001. (F) PCA plot showing the endogenous DNA methylation pattern of the whole genome in the adult human heart. Circles indicate atrium samples, and triangles indicate ventricle samples. The variation values of PC1 and PC2 were 7.4% and 5.5%, respectively. (G) GO terms of genes corresponding to gene bodies in which DMRs were located. Top, GO terms corresponding to DMRs with higher endogenous DNA methylation levels in the atria; bottom, GO terms corresponding to DMRs with higher endogenous DNA methylation levels in the ventricles. (H) PCA plot showing the endogenous DNA methylation pattern of promoters in the adult human heart. Circles indicate atrium samples, and triangles indicate ventricle samples. The variation values of PC1 and PC2 were 8.4% and 7.4%, respectively. (I) Heatmaps showing endogenous DNA methylation levels around the gene body ± 10 kb regions (left) and boxplots showing the gene expression levels (right) of *MYL2* and *MYL3* in the adult human heart. The color bars in the heatmaps indicate the gene-body regions and the promoter regions (from 1 kb upstream of the TSS to 0.5 kb downstream of the TSS). Gene expression levels were quantified with log_2_(RPKM + 1). The raw data for A–I can be found in S1 Data. DEGs, differentially expressed genes; DMRs, differentially methylated regions; GO, Gene Ontology; LA, left atrium; lncRNAs, long noncoding RNAs; LV, left ventricle; PCA, principal component analysis; RA, right atrium; RPKM, reads per kilobase per million; RV, right ventricle; TSS, transcription start site.

We first analyzed the transcriptomes of the 4 chambers of the adult human heart. Notably, principal component analysis (PCA) clearly distinguished between the atria and the ventricles (Fig 2A). However, individual atrium (LA, RA) samples or ventricle (LV, RV) samples were almost indistinguishable. A substantial number of protein-coding genes were differentially expressed in the human atria and ventricles (S2 Table). A total of 808 differentially expressed genes (DEGs) highly expressed in the atria were mainly relevant to the Gene Ontology (GO) terms including cell adhesion, extracellular matrix organization, or ion transport. In addition, the 381 DEGs highly expressed in the ventricles were enriched in the GO terms including oxidation–reduction process, blood circulation, and heart contraction (Fig 2B). Long noncoding RNAs (lncRNAs) have emerged as important regulators of cardiac development and homeostasis [37–40]. Our results showed that PCA based on lncRNA transcriptome data also distinguished between the atria and the ventricles (Fig 2C). We identified 124 and 63 lncRNAs with significantly higher expression in the atria and ventricles, respectively (S2A Fig, S2 Table). Pearson analysis found a statistically significant correlation between these lncRNAs and protein-coding genes compared with random pairs (Fig 2D). Then, we identified 19 and 5 gene pairs that showed *cis*-regulatory relationships between lncRNAs and protein-coding genes in the atria and the ventricles, respectively (Fig 2E). Among these gene pairs, *NKX2-6* is known to play a key role in cardiac development, and its mutation has been linked to familial atrial fibrillation [41,42]; *KCNJ3* is a causative gene of hereditary bradyarrhythmias [43]; *MYL2* has been reported to be connected to hypertrophic cardiomyopathy [44,45]; and *HEY2* participates in left ventricular maturation [46]. Among the lncRNAs in these gene pairs, *CRNDE*, *CHL1-AS2*, and *TBX5-AS1* have been associated with different types of cancers [47–49]. The roles of the lncRNAs in these gene pairs in cardiac development and homeostasis remain to be further investigated.

In our study, to assess the methylation levels of over 16 million WCG sites, we divided the whole genome into 1-kb bins and, notably, found that the atria and the ventricles could be clearly distinguished (Fig 2F). Then, differentially methylated regions (DMRs) between the human atria and ventricles were identified (S3 Table). There were 278 DMRs hypermethylated in the atria, and GO analysis showed that the genes associated with these DMRs were enriched in the GO terms such as contraction and cardiac chamber morphogenesis; 1,199 DMRs were hypermethylated in the ventricles, and they were highly enriched in the GO terms such as cell adhesion and heart development (Fig 2G). While these DMRs tended to occur in the intergenic regions, a number of DMRs were located in promoter and CpG island (CGI) regions (S2B Fig). PCA based on DNA methylation levels in both the promoter and CGI regions distinguished the atria and ventricles (Fig 2H, S2C Fig). Interestingly, the methylation levels in both the promoters and gene-body regions of *MYL2* and *MYL3* were notably lower in the ventricles than in the atria (Fig 2I). The gene body of *MYH7* also showed lower methylation levels in the ventricles (S2D Fig). These 3 genes are all typical ventricle marker genes, and their mutations can lead to hypertrophic cardiomyopathy [45,50,51]. Three genes highly expressed in the atria, *GJA5*, *SMAD6*, and *SMAD7*, showed an opposite methylation pattern, with lower DNA methylation levels in their gene bodies in the atria than in the ventricles (S2D Fig). These genes also contribute to heart development or functions [52–54]. Thus, our results identified a negative correlation between gene expression and gene-body methylation in several key heart genes.

Chromatin accessibility is a prerequisite for transcription factor (TF) binding and can regulate gene transcription [55]. To map open chromatin regions, we identified 766,486 distal nucleosome-depleted regions (NDRs) and 33,546 proximal NDRs in the human heart. Based on the chromatin accessibility level of the distal or proximal NDRs, the 4 chambers of the human heart could not be distinguished from each other (S2E Fig), suggesting that there were no significant differences in the overall chromatin accessibility in the 4 chambers of the heart.

### Comparison of fetal and adult human hearts

**Fig 3:**
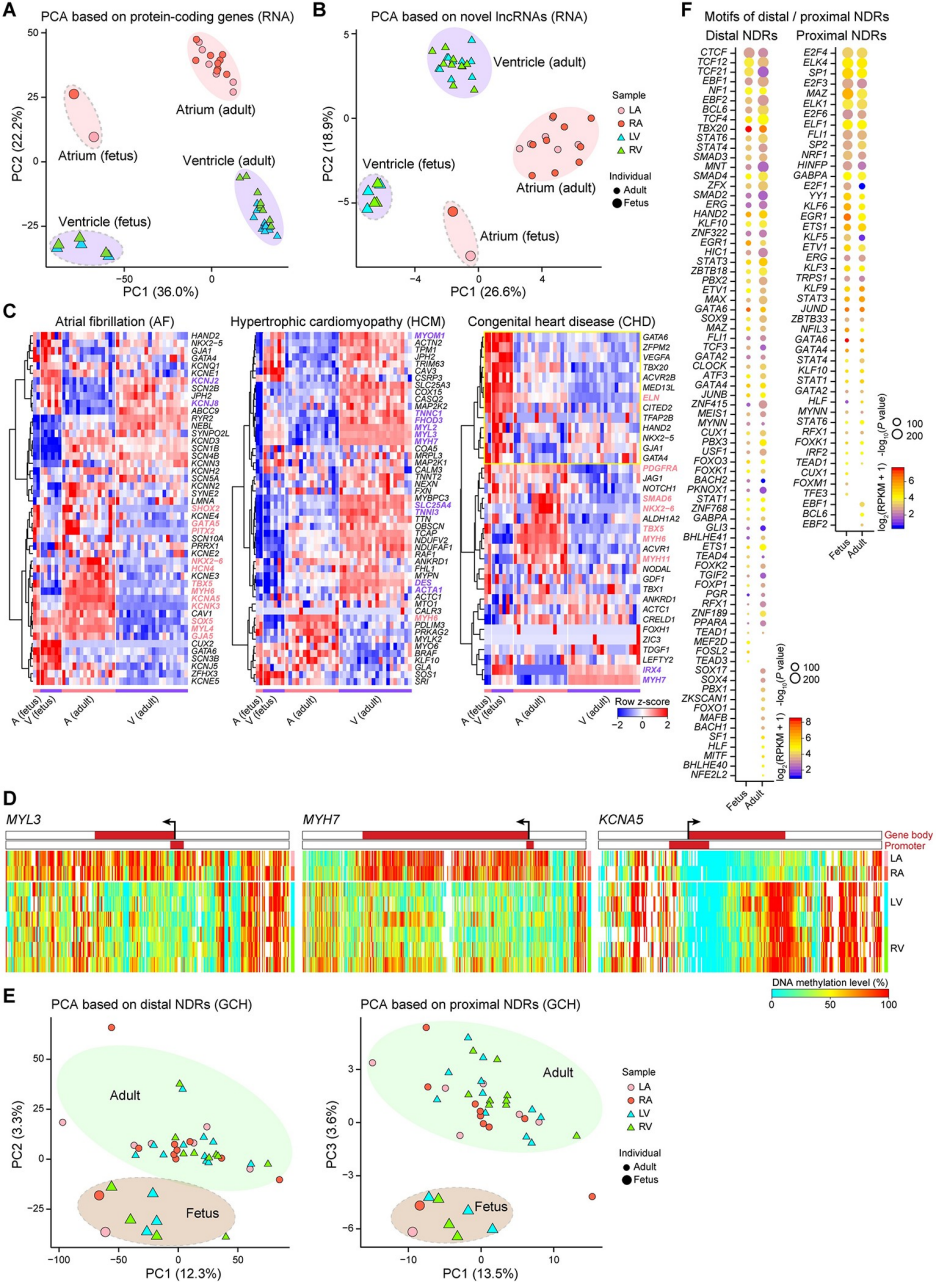
Distinct features of the transcriptome, endogenous DNA methylation, and chromatin accessibility in the human heart between fetuses and adults. (A) PCA plot showing the transcriptome pattern of protein-coding genes in the human heart. Circles and triangles indicate the atria and ventricles, respectively; point sizes indicate the adult heart and fetal heart. The variation values of PC1 and PC2 were 36.0% and 22.2%, respectively. (B) PCA plot showing the transcriptome pattern of novel lncRNAs in the human heart. Circles and triangles indicate the atria and ventricles, respectively; point sizes indicate the adult heart and fetal heart. The variation values of PC1 and PC2 were 26.6% and 18.9%, respectively. (C) Heatmaps showing *z*-score scaled expression levels of genes associated with heart diseases, such as atrial fibrillation (left), hypertrophic cardiomyopathy (middle), and congenital heart disease (right) in the rows. DEGs between the atria and ventricles among these genes are indicated by pink and purple. (D) Heatmaps showing endogenous DNA methylation levels in the gene body ± 10 kb regions of *MYL3* (left), *MYH7* (middle), and *KCNA5* (right) in the human fetal heart. The color bars in the heatmaps indicate the gene-body regions and the promoter regions (from 1 kb upstream of the TSS to 0.5 kb downstream of the TSS). (E) PCA plot showing the chromatin accessibility pattern of distal (left) and proximal (right) NDRs in the human heart. Circles and triangles indicate the atria and ventricles, respectively; point sizes indicate the adult heart and fetal heart. (F) Motif enrichment analysis of distal (left) and proximal (right) NDRs in the human heart. Colors indicate average expression levels, and sizes indicate the *P* values (*P* ≤ 10^−10^) of the corresponding transcription factors. The raw data for A–F can be found in S1 Data. A, atrium; AF, atrial fibrillation; CHD, congenital heart disease; DEGs, differentially expressed genes; HCM, hypertrophic cardiomyopathy; LA, left atrium; lncRNAs, long noncoding RNAs; LV, left ventricle; NDRs, nucleosome-depleted regions; PCA, principal component analysis; RA, right atrium; RPKM, reads per kilobase per million; RV, right ventricle; TSS, transcription start site; V, ventricle.

Then, we compared the fetal and adult human heart. PCA using transcriptome data of both protein-coding genes and known lncRNAs showed that the PC1 axis separated the samples by developmental stage and the PC2 axis distinguished between the atria and the ventricles (Fig 3A, S3A Fig). We identified 176 novel lncRNAs in the human heart (S4 Table, see Methods). The distance from these novel lncRNAs to the nearest known transcripts was 33 kb, and their median nearest distance from each other was 283 kb (S3B Fig). Interestingly, PCA using transcriptome data of these 176 novel lncRNAs could distinguish between the atria and the ventricles of both fetal and adult human hearts, suggesting that they may play roles in heart development (Fig 3B).

Cardiovascular disease is the top killer of humans [56]. Atrial fibrillation (AF), hypertrophic cardiomyopathy (HCM), and congenital heart disease (CHD) are common cardiovascular diseases, and genes associated with these diseases have been uncovered in recent years [57–59]. Here, we displayed the expression patterns of AF-associated genes (47 genes), HCM-associated genes (50 genes), and CHD-associated genes (35 genes) in fetal and adult human hearts (Fig 3C). AF is characterized by abnormal electrical activity of the atrium, and HCM is characterized by thickening of the left ventricular wall [60,61]. We then investigated the overlap distribution between highly expressed DEGs in atria/ventricles and disease-associated genes. We found that there were 14 DEGs overlapped with AF-associated genes, including 12 (85.7%) DEGs were highly expressed in the atria (i.e., *SHOX2*, *GATA2*, and *PITX2*). And there were 11 DEGs overlapped with HCM-associated genes, including 10 (90.9%) DEGs were highly expressed in the ventricles (i.e., *MYOM1*, *TNNC1*, and *DES*). The percentage of DEGs up-regulated in the corresponding chambers implied that chamber-specific expression patterns contributed to disease occurrence and progression (S3C Fig). CHD is a congenital cardiovascular disease related to abnormal heart structure and function [62]. Thirteen CHD-associated genes showed relatively higher expression in fetal hearts (yellow box highlight in Fig 3C). The regional and temporal specificity of cardiovascular disease-associated genes demonstrated that the physiology and pathology of the human heart are under precise spatiotemporal expression and regulation. To reveal potential core genes in cardiovascular diseases, we also constructed coexpression networks of disease-associated genes (S3D Fig). Coexpression networks showed several key genes in these cardiovascular diseases, for example, *KCNK3*, *TNNI3*, and *ELN*.

Considering the DNA methylome, the adult and fetal hearts could be clearly distinguished by the whole genome, promoters, or CGI data. The DNA methylation levels of the whole genome, but not those of promoters or CGI, could distinguish between the atria and the ventricles of fetal hearts (S3E Fig). The gene-body methylation levels of *MYL2*, *MYL3*, and *MYH7* were lower in the fetal ventricles than in the fetal atria, similar to those of the adult heart (Fig 3D). The gene-body methylation level of *KCNA5* was significantly higher in the ventricles than in the atria of fetal hearts, while no difference was found in the adult hearts (Fig 3D).

We identified 886,426 distal NDRs and 34,700 proximal NDRs by combining the adult and fetal human heart data. Notably, PCA using the chromatin accessibility of the proximal or distal NDRs clearly distinguished between fetal and adult hearts (Fig 3E). The enriched TF motifs of the distal and proximal NDRs of fetal and adult hearts were compared (S5 Table). The fetal and adult human heart shared most of the enriched distal motifs, such as the GATA factors (GATA2, GATA4, and GATA6), STAT proteins (STAT1, STAT3, STAT4, and STAT6), and PBX2/3, though not PBX1 (Fig 3F). The binding motifs of a few TFs, such as MITF, which has been shown to play a role in cardiac growth and hypertrophy [63], displayed adult-specific enrichment (Fig 3F). The proximal NDRs in both fetal and adult hearts were strongly enriched for the motifs of TFs related to basic transcription regulation.

### Comparison of human and mouse hearts

**Fig 4:**
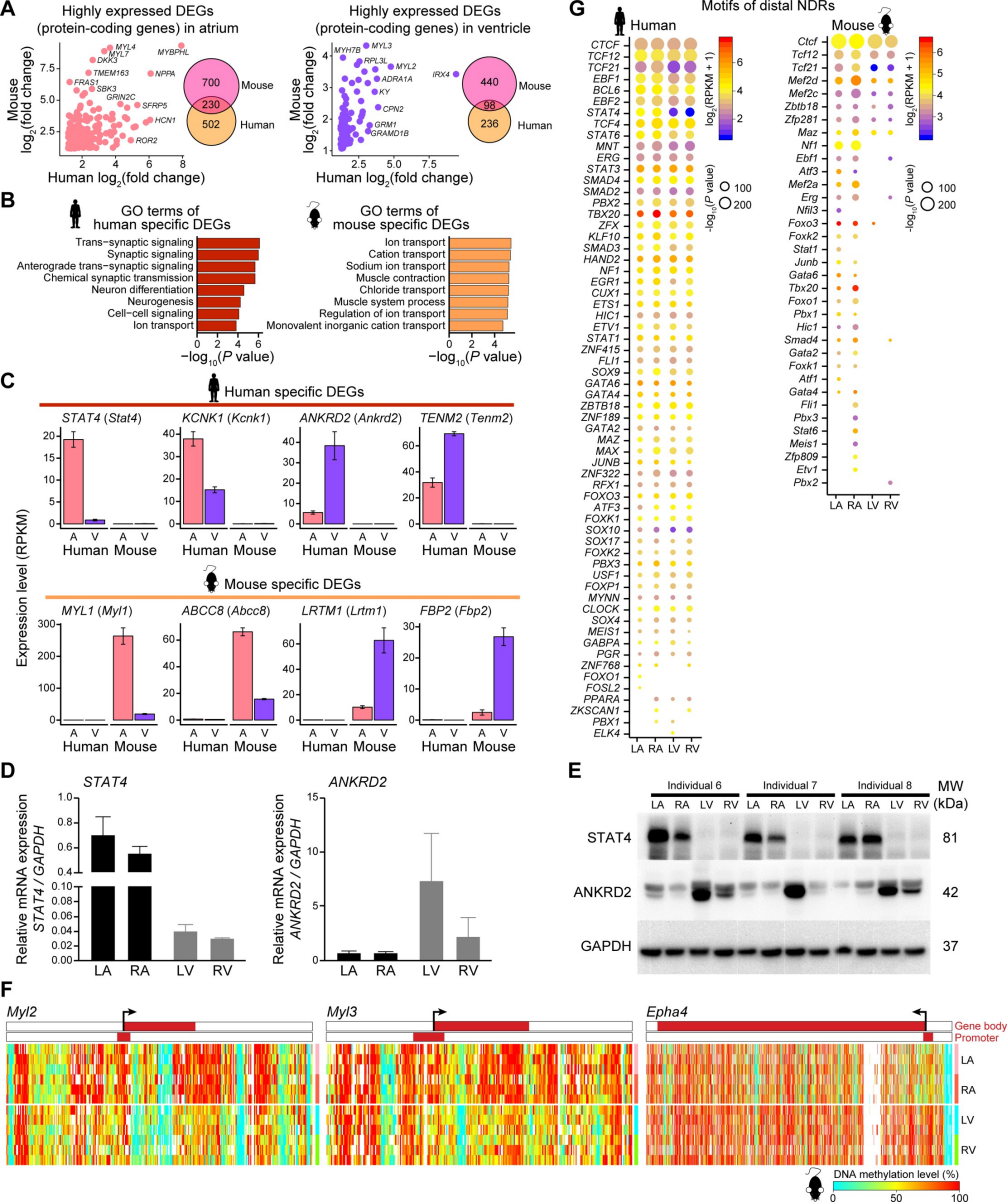
Comparisons of the transcriptome and regulatory elements in the adult human heart and mouse heart. (A) Scatter plots showing fold change of the overlapping DEGs (protein-coding genes) in cross-species comparisons. Venn diagrams showing the number of overlapping DEGs (protein-coding genes) in cross-species comparisons. DEGs were defined as differentially expressed genes with statistical significance between the atria and ventricles. Left, highly expressed DEGs (protein-coding genes) in the atria; right, highly expressed DEGs (protein-coding genes) in the ventricles. (B) GO terms of human- (left) and mouse- (right) specific DEGs. DEGs were defined as in Fig 4A. (C) Bar plots showing the expression levels of human- (top) and mouse- (bottom) specific DEGs. DEGs were defined as in Fig 4A. Data are shown as the mean ± SEM. (D) Relative mRNA levels of *STAT4* and *ANKRD2* in the 4 chambers of the human heart using quantitative RT-PCR. (E) Protein abundances of STAT4 and ANKRD2 in the 4 chambers of the human heart by western blot analysis. (F) Heatmaps showing endogenous DNA methylation levels in the gene body ± 10 kb regions of *Myl2* (left), *Myl3* (middle), and *Epha4* (right) in mouse hearts. The color bars in the heatmaps indicate the gene-body regions and the promoter regions (from 1 kb upstream of the TSS to 0.5 kb downstream of the TSS). (G) Motif enrichment analysis of distal NDRs in the adult human heart (left) and mouse heart (right). Colors indicate average expression levels, and sizes indicate the *P* values (*P* ≤ 10^−10^) of the corresponding transcription factors. The raw data for A–D, F, and G can be found in S1 Data. The raw images for E can be found in S1 Raw images. A, atrium; DEGs, differentially expressed genes; GO, Gene Ontology; LA, left atrium; LV, left ventricle; MW, molecular weight; NDRs, nucleosome-depleted regions; RA, right atrium; RPKM, reads per kilobase per million; RT-PCR, reverse transcription PCR; RV, right ventricle; TSS, transcription start site; V, ventricle.

Next, we performed a comparative analysis of human and mouse hearts. DEGs between the atria and the ventricles of the murine heart were enriched in GO terms such as cell adhesion, heart development, and cardiac muscle contraction, which were highly similar to those of the human heart (S4A Fig, S6 Table). The human and mouse hearts shared 230 DEGs with higher expression levels in the atria, including *NPPA* and *ROR2*, and 98 DEGs with higher expression levels in the ventricles, including *MYL2* and *MYL3* (Fig 4A, S6 Table). We further investigated species-specific DEGs that showed over 5-fold changes in average expression levels and were not detected (reads per kilobase per million (RPKM) < 1) in the other species. In total, 199 human-specific DEGs were identified and found to be enriched in the GO terms such as synaptic signaling and neurogenesis (Fig 4B, S6 Table), while 150 mouse-specific DEGs were revealed and shown to be related to ion transport. *STAT1* and *STAT3* have been shown to play roles in ischemic heart disease [64,65], and in our study, we found that a gene in the STAT family (*STAT4*) was a human-specific DEG (Fig 4C). *ANKRD2* was detected in the adult human heart and has been determined to be up-regulated in human dilated cardiomyopathy [66]. The expression patterns of these genes were confirmed by real-time quantitative reverse transcription PCR (qRT-PCR) and western blotting analysis (Fig 4D and 4E). Compared with single-cell RNA-seq data sets of human and mouse hearts [16,67], we found that both human-specific and mouse-specific ventricular DEGs (with higher expression levels in the ventricles) were mainly expressed in cardiomyocyte (S4B and S4C Fig). In the meantime, those species-specific atrial DEGs (with higher expression levels in the atria) were expressed in several cell types, such as fibroblast, neuron, endothelial cell, and cardiomyocyte (S4B and S4C Fig). In mouse hearts, PCA based on DNA methylation of the whole genome, only the promoters or only the CGI regions distinguished the atria from the ventricles, similar to the results in human hearts (S4D Fig). The gene bodies of *Myl2* and *Myl3* were also hypermethylated in the mouse atria compared with the ventricles (Fig 4F). In addition, *Epha4* showed a higher methylation level in the gene body in the ventricles, and this phenomenon was conserved in both mouse and human hearts (Fig 4F, S4E Fig).

TF binding motif enrichment analysis was performed on the NDRs to establish the relationship between TF regulation and open chromatin. To compare motif enrichment related to chromatin accessibility between human and mouse hearts, we identified 313,517 distal NDRs and 25,966 proximal NDRs in the mouse heart to seek motifs for TF binding. The results showed that the distal NDRs of both species were enriched for the binding motifs of TFs such as GATAs (GATA2, GATA4, and GATA6), STATs (STAT1 and STAT6), and PBX1/2/3 (Fig 4G, S7 Table). GATA factors play important roles in heart development and cardiac diseases, and overexpression of *Gata4* or *Gata6* in mouse hearts could lead to cardiac hypertrophy [68,69]. Pbx proteins are necessary for myocardium differentiation [70]. Tcf21 functions in specification of the cardiac fibroblast lineage as a bHLH TF [71] and showed higher expression in the atria. These key TFs formed conserved regulatory networks in both human and mouse hearts. We also identified binding motifs for a few TFs that were enriched solely in either human or mouse hearts. Mef2 is an early marker of the myocardium lineage [72]. Interestingly, although *MEF2A* (mean expression level RPKM = 46), *MEF2C* (RPKM = 13), and *MEF2D* (RPKM = 29) were expressed in human hearts, the binding motifs of Mef2a, Mef2c, and Mef2d were enriched in only mouse hearts. The proximal NDRs of both human and mouse hearts were enriched in the motifs of TFs such as Sp proteins (SP1 and SP2), the E2F TF family (E2F4 and E2F6), and Kruppel-like factors (KLF3, KLF6, and KLF9), which are mainly involved in basic transcription regulation (S4F Fig, S7 Table).

### Correlations among DNA methylation, chromatin accessibility, and gene expression

**Fig 5:**
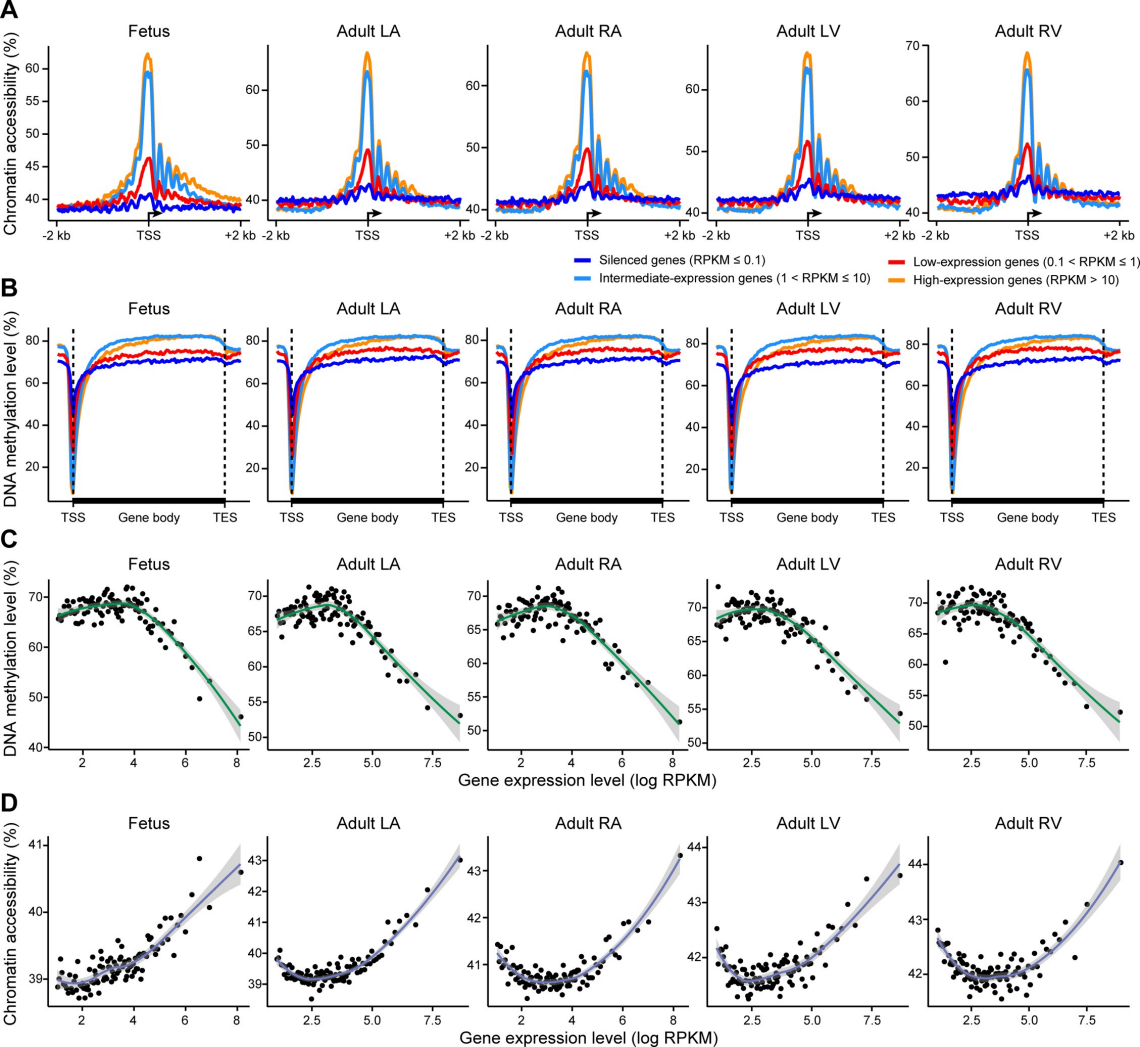
Multiomics relationship patterns in the human heart. (A and B) Line plots showing the average chromatin accessibility level in the TSS ± 2 kb regions (A) and the average endogenous DNA methylation level in the gene body ± 5 kb regions (B) of protein-coding genes in the human heart. Different colors indicate 4 gene groups classified according to expression levels. (C and D) Expressed genes (RPKM > 0) were ranked by expression level and arranged into 100 bins, and the x-axis from left to right represents genes with increased expression levels. Each point represents a bin. The line plots show the average endogenous DNA methylation level (C) and the average chromatin accessibility level (D) of the gene bodies in each bin. The shadow indicates the 0.95 confidence interval around smooth. The raw data for A–D can be found in S1 Data. LA, left atrium; LV, left ventricle; RA, right atrium; RPKM, reads per kilobase per million; RV, right ventricle; TES, transcription end site; TSS, transcription start site.

To explore how DNA methylation and chromatin accessibility are correlated with gene expression, all the protein-coding genes were divided into 4 groups: high- (RPKM > 10), intermediate- (1 < RPKM ≤ 10), and low-expression genes (0.1 < RPKM ≤ 1) and silenced genes (RPKM ≤ 0.1). We first observed the average chromatin accessibility levels around the TSS and found that chromatin accessibility was positively correlated with gene expression levels. The high-expression genes showed the highest level of accessibility, and the silenced genes were nearly inaccessible (Fig 5A). The high-expression lncRNAs also showed higher accessibility around the TSS than the low-expression lncRNAs (S5A Fig).

Then, we investigated the average DNA methylation levels across the genes. Consistent with previous reports, DNA methylation around the TSS was negatively correlated with gene expression (Fig 5B, S5B Fig). In promoter regions, we found a clear positive correlation (R1) between the expression levels of protein-coding genes and the corresponding chromatin accessibility level (S5C Fig). In contrast, a negative correlation (R2) between expression and DNA methylation level was observed.

Interestingly, the results showed that the gene bodies of intermediate-expression genes exhibited the highest methylation level (Fig 5B). Indeed, gene-body methylation displayed a bell-shaped relationship with gene expression, with high-expression genes showing prominently lower methylation levels than intermediate-expression genes (Fig 5C). Notably, gene-body chromatin accessibility revealed a reverse bell-shaped relationship with gene expression, indicating that the gene-body chromatin of high-expression genes was generally open in the human heart (Fig 5D). These patterns of correlations between gene expression and DNA methylation or chromatin accessibility were conserved in mice (S5D and S5E Fig).

## Discussion

This study, to our knowledge, represents the first comprehensive multiomics analysis of all 4 chambers of adult human and mouse hearts and fetal human hearts. The mouse is the most widely used model for studying heart development and cardiac diseases. Although human and mouse hearts are similar in anatomy, they are quite different in size and electrophysiology [2,73–76]. Additionally, studies have shown that human and mouse hearts respond differently to cardiovascular drugs [77,78]. Our data showed that the NDRs of human and mouse hearts share many TF motifs, indicating that the main regulatory networks are conserved between these 2 species. On the other hand, the species-specific DEGs and motifs identified in our data provide insights into the species differences between human and mouse hearts that could potentially accelerate the development of cardiovascular drugs. A recent study revealed transcriptional and cellular diversity of the nonfailing human heart at single-cell resolution and identified a cluster of neuronal cells which were likely to derived from the intrinsic cardiac autonomic network [16]. The identified human-specific DEGs in our study related to synaptic signaling showed relatively higher expression level in neuronal cells than other types of cells.

Our study revealed significant differences among 4 chambers of the adult human heart and between fetal and adult human hearts. We identified novel chamber-specific and stage-specific lncRNAs, as well as lncRNAs that were paired with important genes, suggesting that they are worth further research. The epigenomes of the different chambers and developmental stages were distinguishable, reflecting the roles of epigenetic modifications in precise spatiotemporal regulation of heart development and cardiac homeostasis. Some key ventricle marker genes, such as *MYL2*, *MYL3*, and *MYH7*, showed gene-body hypomethylation in the ventricles in both the fetal and adult stages, suggesting that there are critical regulatory elements in these regions.

The complex interactions characterized by multiomics guide diverse cells and organs to play their roles. A negative correlation between gene expression and DNA methylation with positively correlated gene expression and chromatin accessibility has been reported in mouse embryonic stem cells using a single-cell multiomics method [79]. While our data are largely consistent with this study, we interestingly found that the gene bodies of intermediate-expression genes in the human heart had the highest level of DNA methylation and lowest level of chromatin accessibility. A previous study also showed a bell-shaped correlation between gene expression and gene-body DNA methylation in cell lines [80]. It is possible that the relatively high transcription rate of the intermediate-expression genes combined with the presence of few intragenic regulatory elements contributes to this pattern. The detailed mechanism underlying the bell-shaped relationship between expression and gene-body DNA methylation or chromatin accessibility needs further research in the future.

In summary, our study provides integrated transcriptome, DNA methylation, and chromatin accessibility maps for understanding the spatiotemporal characteristics of both the human and mouse hearts.

## Methods

### Heart sample collection

Samples of normal adult human hearts were obtained from donors who died for reasons other than cardiovascular disease (*n =* 11; 10 male; mean age, 29.4 ± 7.1 years). Human fetal hearts aged 16 (*n* = 1) and 20 weeks (*n* = 2) of gestation were collected after elective abortion. The demographics of the donors are summarized in S1 Table. The LA, RA, LV, and RV were collected and immediately frozen in liquid nitrogen for future use as soon as adult or fetal hearts were separated. This study was performed in accordance with the principle of the Helsinki Declaration and approved by the Ethics Committees of Fuwai Hospital and Xiangyang No.1 People’s Hospital. The approval numbers for adult and fetal human samples were 2018–1065 and XYFH20170124, respectively. Written informed consent was obtained from all donors or parents. Samples of all 4 cardiac chambers from C57BL/6 mice aged 10 weeks were collected for cross-species analysis, in conformity with the Chinese National Regulation on the Administration of Laboratory Animals (2017 Revision) and approved by the Fuwai Hospital Committee for Laboratory Animal Use (approval number: FW-2019-0002). To avoid gender-related effects in the cardiovascular system of mice [81–84], the mice used in this study were all male.

### RNA extraction and RNA-seq library preparation

Following sample homogenization by grinding in liquid nitrogen and subsequent use of QIAshredder (QIAGEN, Dusseldorf, North Rhine-Westphalia, Germany, Cat. 79656), total RNA from human and mouse hearts was extracted using the RNeasy Fibrous Tissue Mini Kit (QIAGEN, Cat. 74704) according to the manufacturer’s recommendation. Then, rRNAs were removed from the total RNA by the NEBNext rRNA Depletion Kit (NEB, Ipswich, Massachusetts, USA, Cat. E7755X). First-strand cDNAs were synthesized by the NEBNext RNA First Strand Synthesis Module (NEB, Cat. E7525L), and second-strand synthesis was performed by the NEBNext Ultra II Non-Directional RNA Second Strand Synthesis Module (NEB, Cat. E6111L). Finally, the RNA-seq library was prepared using KAPA Hyper Prep Kits (KAPA Biosystems, Wilmington, Massachusetts, USA, Cat. KK8504).

### NOMe-seq library preparation and sequencing

To optimize the NOMe-seq procedure for frozen heart tissue samples, we modified the protocol based on previous studies [29,34,85,86]. Approximately 30 mg tissues were used for NOMe-seq in this study. In brief, the heart tissues were thoroughly ground at low temperatures. The tissue pellets were resuspended in 500 μl ice-cold lysis buffer with Protease Inhibitor Cocktail: 10 mM Tris·HCl (pH 7.5), 10 mM NaCl, 3 mM MgCl_2_, 0.1 mM EDTA, and 0.5% NP-40. The tubes were incubated for 60 min with vortexing every 10 min to release nuclei. The nuclei were washed twice with cold DPBS, and the lysates were incubated in 60 units of M.CviPI GpC Methyltransferase (NEB, Cat. M0227L) for 1 h, with the addition of 3 ng unmodified lambda DNA (Thermo Fisher Scientific, Waltham, Massachusetts, Cat. SD0021). Twenty units of M.CviPI and 0.75 μl of 200× SAM were added for another 1 h incubation. The reactions were then stopped by adding EDTA and proteinase K to digest the proteins overnight. Genomic DNAs were purified by phenol:chloroform:isoamyl alcohol extraction and ethanol precipitation. Next, bisulfite conversion of genomic DNAs was conducted using EZ-96 DNA Methylation-Direct MagPrep (Zymo Research, Irvine, California, USA, Cat. D5044). Oligo1 (5′-biotin- CTACACGACGCTCTTCCGATCTNNNNNNNNN-3′) and Oligo2 (AGACGTGTGCTCTTCCGATCTNNNNNNNNN) were used to synthesize the first and second strands, respectively. Finally, the NOMe-seq libraries were amplified with 12 cycles of PCR using KAPA HiFi Hot Start Ready Mix (KAPA Biosystems, Cat. KK2602). The NOMe-seq libraries and RNA-seq libraries were sequenced on the HiSeq 4000 platform (Novogene, Beijing, China).

### Quantitative RT-PCR

Total RNA was isolated using TRIzol (Thermo Fisher Scientific, Cat. 15596018) according to the manufacturer’s protocol, and reverse transcription was performed with the PrimeScript RT reagent Kit (Takara, Beijing, China, Cat. RR047A). Quantitative PCR was performed in an ABI 7500 using TB Green Premix Ex Taq (Takara, Cat. RR820B). After an initial denaturation at 95°C for 30 s, the reaction conditions (5 s at 95°C, 34 s at 60°C, 34 s at 72°C) were applied for 40 cycles, and each qRT-PCR was repeated thrice for every sample as technical replicates. Relative mRNA expression was calculated by the 2^-△△CT^ method and normalized using *GAPDH* as an internal control. The specific primers used were as follows: for human *STAT4*, forward primer 5′-ATCCTCCACCTGCCACATTG-3′ and reverse primer 5′-TTCCTTGCAGAACTTGGCCC-3′; for human *ANKRD2*, forward primer 5′-AGACCTTCCTGAAAGCTGCG-3′ and reverse primer 5′-TCCACAGTGGCCCCATTATC-3′; for human *GAPDH*, forward primer 5′-ACAACTTTGGTATCGTGGAAGG-3′ and reverse primer 5′-GCCATCACGCCACAGTTTC-3′.

### Western blot analysis

Total proteins were harvested using RIPA buffer supplemented with proteinase and phosphatase inhibitors (Roche, Basel, Switzerland, Cat. 05892791001, Cat. 04906845001) according to standard protocols. The protein concentrations were measured by the BCA (Thermo Fisher Scientific, Cat. 23227) assay. The protein samples were separated by 4% to 12% SDS-PAGE gels (Invitrogen, Carlsbad, California, USA, Cat. NP0323BOX) followed by electrotransfer to polyvinylidene fluoride (PVDF) membranes (Merck Millipore, Burlington, Massachusetts, USA, Cat. ISEQ00010). The membranes were blocked in 5% milk in Tris-buffered saline (TBS)-Tween (Applygen, Beijing, China, Cat. B1009) for 1 h at room temperature and then incubated overnight at 4°C in primary antibodies against STAT4 (CST, Danvers, Massachusetts, USA, Cat. 2653, 1:1,000), ANKRD2 (Proteintech, Rosemont, Illinois, USA, Cat. 11821, 1:1,000), or GAPDH (Proteintech, Cat. 60004, 1:20,000). After the membranes were washed 5 times in TBS-T for 5 min at room temperature, they were incubated with secondary antibodies conjugated to HRP (ZSGB-BIO, Beijing, China, Cat. ZB-2301, Cat. ZB-2305) for 1 h at room temperature. After washing 5 times in TBS-T for 5 min, protein bands were visualized using the SuperSignal West Femto Chemiluminescent Substrate Kit (Thermo Fisher Scientific, Cat. 34096) and imaged using the ProteinSimple FluorChem M System (ProteinSimple, San Jose, California, USA).

### Processing NOMe-seq data

The NOMe-seq raw sequencing reads were processed by removing the first 9 bases containing random primer sequences, adaptors, and low-quality bases using *trim_galore* (version: 0.1.3) with the parameters ‘*—quality 20—stringency 3—length 50—clip_R1 9—clip_R2 9—paired—trim1—phred33*’. The clean reads were then mapped into the UCSC human genome (hg19) or mouse genome (mm10) using *Bismark* (version: 0.7.6) with the parameters ‘*—fastq—non_directional—unmapped—phred33-quals*’ in the paired-end nondirectional alignment mode [87]. Then, the unmapped reads were realigned into the same reference genome in a single-end alignment mode to improve the number of mapped reads. After alignment, PCR-duplicated reads were removed using *SAMtools* (version: 0.1.18) [88], and the final BAM file was obtained. Cytosine sites with at least 3× coverage were used to detect technological replicates. After that, the final BAM files of the same technological replicate were merged using *SAMtools*, and 3× coverage was used as the read depth cutoff in the downstream analysis.

The methylation level of each cytosine site was calculated as the number of methylated reads ‘C’ divided by the number of methylated and unmethylated reads (‘C+T’). We used WCG (ACG and TCG trinucleotides) for DNA methylation analysis and GCH (GCA, GCT, and GCC trinucleotides) for chromatin accessibility analysis.

### Processing RNA-seq data

RNA-seq raw sequencing reads were first processed by discarding reads contaminated with adaptors and reads with low-quality bases using custom scripts. Then, the clean reads were mapped to the GENCODE human genome (hg19) or mouse genome (mm10) using *HISAT2* (version: 2.1.0) [89] with the default parameters. We generated splice site files for mapping with the *hisat2_extract_splice_sites*.*py* script in *HISAT2*. In each sample, the transcripts were then directly assembled and quantified using the subcommand ‘*-e -B*’ in *StringTie* (version: 1.3.6) [90] with read alignments and the reference annotation. Finally, the gene expression levels were quantified as reads per kilobase per million mapped reads (RPKM).

### Identification of differentially expressed genes

DEGs were identified with the RNA-seq read count data using the R package *DESeq2* (version: 1.20.0) [91]. The false discovery rate (FDR, Benjamini and Hochberg) was calculated to obtain the statistical significance of DEGs. Only genes matching the following 2 criteria were considered DEGs: (1) the log2-transformed fold change was greater than 1 with a *P* value ≤ 0.05 and FDR ≤ 0.05; (2) the average expression level (RPKM) in the corresponding cluster was greater than 1 (for example, the average expression level of a highly expressed DEG in the atria should be greater than 1 in the atria). GO analysis of the DEGs was performed with ToppGene [92].

### Identification of correlations between long noncoding RNAs and protein-coding RNAs

The correlation between lncRNAs and protein-coding RNAs was calculated with DEGs in the atria and ventricles. There were 808 protein-coding RNAs and 124 lncRNAs in the atria and 381 protein-coding RNAs and 63 lncRNAs in the ventricles. We calculated the Pearson correlation between each lncRNA and any protein-coding RNAs within 500 kb, and there were 67 and 30 such pairs in the atria and ventricles, respectively.

LncRNAs and protein-coding RNAs were then randomly arranged throughout the genome to generate random gene pairs, but the gene length was kept fixed. The Pearson correlation between lncRNAs and protein-coding RNAs within 500 kb in the random distribution was calculated. This simulation process was repeated 1,000 times.

### Identification of *cis*-regulatory relationships between long noncoding RNAs and protein-coding RNAs

Pearson correlations between lncRNAs and protein-coding RNAs were calculated to detect coexpressed gene pairs, and only DEGs in the atria and ventricles were considered. Coexpressed gene pairs were chosen only if the Pearson correlation coefficient was greater than 0.6 and the correlation *P* value was ≤ 0.05. If the linear distance between the lncRNA and protein-coding RNA in the coexpressed gene pair was less than 100 kb, we considered this gene pair to have a *cis*-regulatory relationship.

### Identification of species-specifically expressed genes

We first examined gene homology correspondences from the human and mouse annotation references [93] and discarded genes with one-to-many correspondences. Then, genes with the following 3 features were identified as species-specifically expressed genes (in short, taking human-specifically expressed genes as an example): (1) the average difference in the gene expression level between humans and mice was more than 5 times; (2) at least 2 human samples expressed this gene (RPKM ≥ 0.1) with an average expression level in all human samples of more than 1; and (3) the average expression level in all mouse samples was less than 1. Finally, 768 human- and 399 mouse-specifically expressed genes were identified. We then overlapped these species-specifically expressed genes with the DEGs in the atria or ventricles to obtain species-specific DEGs.

### Identification of novel transcripts in human hearts

To investigate novel transcripts in humans, the clean RNA-seq sequencing data were aligned with *HISAT2* as described above, but the alignment reads were not directly assembled and quantified using the subcommand ‘*-e -B*’ in *StringTie* with the reference annotation. After the alignment, the transcripts were initially assembled and quantified with *StringTie* in each sample, and then, we merged the transcripts in each human sample with the subcommand ‘*merge*’ in *StringTie*, which created a uniform transcript set for all human samples. We then processed the alignment reads and the merged transcripts using the subcommand ‘*-e -B*’ in *StringTie*.

To exclude known transcripts and identify novel transcripts, annotation files from UCSC, Ensembl, and GENCODE were collected together to form a set of “known transcript sites.” Any transcript located within 10 kb upstream and 10 kb downstream from a known transcript was not defined as a novel transcript. Transcripts not mapped to assembled chromosomes were excluded from the downstream analysis.

After that, 2,424 novel transcripts were obtained, and their coding potential was calculated with the *CPC* (Coding Potential Calculator) online tool [94], which separated these transcripts into 2 groups, coding transcripts and noncoding transcripts. Furthermore, we filtered the novel transcripts with the following 3 criteria: (1) the length of a novel transcript was more than 1 kb; (2) the number of exons in a novel transcript was more than 2 (in other words, there was an exon–exon junction in the transcript); and (3) at least 1 human sample expressed this novel transcript. Finally, 51 coding RNAs and 176 noncoding RNAs were detected. Since the lengths of these filtered noncoding RNAs were more than 1 kb, we directly considered these RNAs as lncRNAs in this study.

### Establishing the coexpression networks with cardiovascular diseases associated genes

To identified the potential key genes in cardiovascular diseases, the coexpression networks analysis was performed only with associated genes. Based on our RNA-seq data quantified with RPKM, the Pearson correlation coefficient was calculated, and only gene pairs with correlation coefficient greater than 0.5 were considered in the following analysis. The networks were visualized with software *Cytoscape*, and the point size indicated the number of connections for a given gene, and the line thickness indicated the correlation coefficient for a given gene pair.

### Estimation of DNA methylation and chromatin accessibility levels

DNA methylation levels (WCG) and chromatin accessibility levels (GCH) were estimated in several different genomic regions, and only regions with at least 3 WCG/GCH sites were considered.

The whole genome (human or mouse) was separated into bins with a size of 1 kb. The gene body was the region from the TSS to the transcription end site (TES). When we observed WCG levels around gene bodies (Fig 1C and 1E, S1A and S1B Fig), we extended the gene-body region (5 kb upstream from TSS and 5 kb downstream from TES), each gene body was separated into 100 bins with different sizes, and each extended region was separated into 10 bins with a size of 500 bp. When we observed GCH levels around the TSS (Fig 1C and 1E, S1A and S1B Fig), we extended the window 2 kb upstream and 2 kb downstream from the TSS, and each region was separated into 200 bins with a size of 20 bp.

The promoter was defined as the region from 1 kb upstream of the TSS to 0.5 kb downstream of the TSS. The human annotation information identifying the genomic elements (exons, introns, and CpG islands) was downloaded from the UCSC Genome Browser (hg19), and all repetitive elements and their subfamilies (LINE, SINE, LTR, L1, L2, Alu, MIR, ERV1, ERVK, ERVL, and ERVL-MaLR) were collected from the “RepeatMasker” track from the UCSC Genome Browser (hg19). Intragenic regions were considered gene-body regions, and intergenic regions were considered the complement of the intragenic regions throughout the genome. Human enhancer information was collected from a previous study [95].

### Identification of differentially methylated regions

DMRs were identified with the 3× coverage WCG sites. The whole genome was separated into several 300 bp fixed windows to calculate the average WCG level in each sample, and windows with at least 3 WCG sites were considered. Differentially methylated windows (DMWs) between 2 group samples were first selected only if the average difference was more than 20% with two-tailed Student *t* test *P* ≤ 0.05 and FDR ≤ 0.05 (Benjamini and Hochberg method). Neighboring DMWs within 300 bp were then joined as DMRs.

### Identification of nucleosome-depleted regions

NDRs were identified with 3× coverage GCH sites. NDRs were defined as regions with significantly higher GCH levels than those in the background. Similar to the previous study [96,97], 100 bp windows with 20 bp sliding steps were used to call NDRs, and only regions matched following 3 criteria were considered NDRs: (1) the average GCH level in the region was significantly higher than that in the whole-genome background with chi-squared test *P* ≤ 10^−10^; (2) the number of GCH sites in the region was more than 5; and (3) the length of the region was longer than 140 bp.

According to the distance from NDRs to TSS, NDRs were grouped into 2 types: proximal NDRs and distal NDRs. NDRs located within promoters (1 kb upstream and 0.5 kb downstream of the TSS) were defined as proximal NDRs, and all others were defined as distal NDRs.

### Identification of transcription factor motifs corresponding to open chromatin

To establish the relationship between TF regulation and open chromatin, TF motif enrichment in NDRs was investigated using the *findMotifsGenome*.*pl* script in *HOMER* (version: 4.10.4) [98] with the parameters ‘*-size 2000 -len 8 -S 100*’. Only motifs with *P* ≤ 10^−10^ and RPKM ≥ 5 in at least 1 subject were kept in the downstream analysis. In the figures of this study, any motifs without statistical significance are not shown.

## Supporting information

S1 Fig Global patterns of DNA methylation and chromatin accessibility in human and mouse hearts. (A) Line plots showing the average endogenous DNA methylation level around the gene body ± 5 kb region (left) and the average chromatin accessibility levels around the TSS ± 2 kb region (right) in the adult human heart NC_LV_ind1. Different colors indicate different technology replications. (B) Line plots showing the average endogenous DNA methylation level around the gene body ± 5 kb region (left) and the average chromatin accessibility levels around the TSS ± 2 kb region (right) in the mouse heart NM_LA_m1to5. Different colors indicate different technology replications. (C) Bar plots showing the average endogenous DNA methylation levels (left) and the average chromatin accessibility levels (right) in the whole genome in the human heart. (D) Bar plots showing the average endogenous DNA methylation levels (left) and the average chromatin accessibility levels (right) in the whole genome in the mouse heart. The raw data for A–D can be found in S2 Data. LA, left atrium; LV, left ventricle; TES, transcription end site; TSS, transcription start site.
(TIF)

S2 Fig Multiomics differences (differences in the transcriptome and endogenous DNA methylation) in the adult human heart. (A) Heatmap showing row *z*-score scaled gene expression levels of DEGs (lncRNAs) between the atria and ventricles in the adult human heart. The number of DEGs is reported on the left. (B) Bar plots showing the relative enrichment of DMRs located in different genome elements. (C) PCA plot showing the endogenous DNA methylation pattern of CpG islands in the adult human heart. Circles indicate atrium samples, and triangles indicate ventricle samples. The variation values of PC1 and PC2 were 10.2% and 7.8%, respectively. (D) Heatmaps showing endogenous DNA methylation levels around the gene body ± 10 kb region of *MYH7* (top), *GJA5*, *SMAD6*, and *SMAD7* (bottom) in the adult human heart. The color bars in the heatmaps indicate the gene-body region and the promoter region (from TSS upstream 1 kb to TSS downstream 0.5 kb). Gene expression levels were quantified with log_2_(RPKM + 1). (E) PCA plot showing the chromatin accessibility pattern of distal (left) and proximal (right) NDRs in the adult human heart. Circles and triangles indicate atrium and ventricle samples, respectively. The raw data for A–E can be found in S2 Data. CGI, CpG island; DEGs, differentially expressed genes; DMRs, differentially methylated regions; LA, left atrium; LINE, long interspersed nuclear elements; lncRNAs, long noncoding RNAs; LTR, long terminal repeat; LV, left ventricle; MIR, mammalian-wide interspersed repeat; NDRs, nucleosome-depleted regions; PCA, principal component analysis; RA, right atrium; RPKM, reads per kilobase per million; RV, right ventricle; SINE, short interspersed nuclear elements; TSS, transcription start site.
(TIF)

S3 Fig Multiomics comparisons (comparisons of the transcriptome, endogenous DNA methylation, and chromatin accessibility) in the human heart between fetuses and adults. (A) PCA plot showing the transcriptome pattern of known lncRNAs in the human heart. Circles and triangles indicate atrium and ventricle samples, respectively; point sizes indicate the adult heart and fetal heart. The variation values of PC1 and PC2 were 24.6% and 16.3%, respectively. (B) Left, line plot showing the density distribution of length from novel transcripts to the nearest known transcripts; the zoom-out visualization of the dashed region is shown. Right, line plot showing the density distribution of the nearest length between each novel transcript. (C) Bar plot showing the distribution of human DEGs in heart disease-associated gene sets. These DEGs were identified with comparisons between the adult atria and ventricles. (D) Coexpression networks of cardiovascular diseases (AF, HCM, and CHD) associated genes. The point size indicated the number of connections for a given gene, and the line thickness indicated the correlation coefficient for a given gene pair. (E) PCA plot showing the endogenous DNA methylation pattern of the whole genome (left), promoters (middle), and CpG islands (right) in the human heart. Rounds and triangles indicate atrium and ventricle samples, respectively; point sizes indicate the adult heart and fetal heart. The raw data for A–E can be found in S2 Data. AF, atrial fibrillation; CHD, congenital heart disease; DEGs, differentially expressed genes; HCM, hypertrophic cardiomyopathy; LA, left atrium; lncRNAs, long noncoding RNAs; LV, left ventricle; PCA, principal component analysis; RA, right atrium; RV, right ventricle.
(TIF)

S4 Fig Comparisons of the transcriptome and regulatory elements in the adult human heart and mouse heart. (A) Heatmap showing row *z*-score scaled gene expression levels of DEGs (protein-coding genes) between the atria and ventricles in the mouse heart (left), and corresponding GO terms are shown (right). The number of DEGs is reported on the left. (B) Heatmaps showing z-score scaled expression levels of human-specific DEGs among different cell types from single-cell RNA-seq data. Left: human-specific DEGs with higher expression levels in the atria; right, human-specific DEGs with higher expression levels in the ventricles. (C) Heatmaps showing z-score scaled expression levels of mouse-specific DEGs among different cell types from single-cell RNA-seq data. Left: mouse-specific DEGs with higher expression levels in the atria; right, mouse-specific DEGs with higher expression levels in the ventricles. (D) PCA plots showing the endogenous DNA methylation pattern of the whole genome, promoters, and CpG islands in the mouse heart. Circles and triangles indicate atrium and ventricle samples, respectively. (E) Heatmaps showing endogenous DNA methylation levels in the gene body ± 10 kb regions of *EPHA4* in human hearts. The color bars in the heatmaps indicate the gene-body regions and the promoter regions (from 1 kb upstream of the TSS to 0.5 kb downstream of the TSS). (F) Motif enrichment analysis of proximal NDRs in the adult human heart (left) and mouse heart (right). Colors indicate average expression levels, and sizes indicate *P* values (*P* ≤ 10^−10^) of the corresponding transcription factor. The raw data for A–F can be found in S2 Data. DEGs, differentially expressed genes; GO, Gene Ontology; LA, left atrium; LV, left ventricle; NDRs, nucleosome-depleted regions; PCA, principal component analysis; RA, right atrium; RPKM, reads per kilobase per million; RV, right ventricle; TSS, transcription start site.
(TIF)

S5 Fig Multiomics relationship patterns in human and mouse hearts. (A and B) Line plots showing the average chromatin accessibility level around the TSS ± 2 kb region (A) and the average endogenous DNA methylation level around the gene body ± 5 kb region (B) of lncRNAs in the human heart. Different colors indicate 2 lncRNA groups classified according to expression levels. (C) Line plots showing the average endogenous DNA methylation level (green) and the average chromatin accessibility level (blue) in promoters of corresponding genes. Genes were ranked by expression levels, and the x-axis from left to right represented genes with increased expression levels. Spearman correlation coefficients between chromatin accessibility levels and gene expression levels (R1) and Spearman correlation coefficients between endogenous DNA methylation levels and gene expression levels (R2) are indicated. (D) Line plots showing the average chromatin accessibility level around the TSS ± 2 kb region (left) and the average endogenous DNA methylation level around the gene body ± 5 kb region (right) of protein-coding genes in the mouse heart LA. Different colors indicate 4 gene groups classified according to expression levels. (E) Line plots showing the average chromatin accessibility level around the TSS ± 2 kb region (left) and the average endogenous DNA methylation level around the gene body ± 5 kb region (right) of lncRNAs in the mouse heart LA. Different colors indicate 2 lncRNA groups classified according to expression levels. The raw data for A–E can be found in S2 Data. LA, left atrium; lncRNAs, long noncoding RNAs; LV, left ventricle; RA, right atrium; RPKM, reads per kilobase per million; RV, right ventricle; TES, transcription end site; TSS, transcription start site.
(TIF)

S1 Table Sequencing information of NOMe-seq and RNA-seq in human and mouse hearts. (XLSX)

S2 Table DEGs (protein-coding genes and lncRNAs) between the human atria and ventricles. (XLSX)

S3 Table DMRs between the human atria and ventricles. (XLSX)

S4 Table Novel lncRNAs identified in human hearts. (XLSX)

S5 Table Motifs enriched in distal and proximal NDRs in fetal and adult human hearts. (XLSX)

S6 Table Conserved and species-specific DEGs between the atria and ventricles in human and mouse hearts. (XLSX)

S7 Table Motifs enriched in distal and proximal NDRs in human and mouse hearts. (XLSX)

S1 Data The individual numerical values in Figs 1B–1E; 2A–2I; 3A–3F; 4A–4D, 4F, 4G; 5A–5D. (XLSX)

S2 Data The individual numerical values in S1A–S1D; S2A–S2E; S3A–S3E; S4A–S4F; S5A–S5E Figs. (XLSX)

S1 Raw images Original gel and blot images for Fig 4E. (PDF)

## Abbreviations

AF: atrial fibrillation
ATAC-seq: assay for transposase-accessible chromatin using sequencing
CGI: CpG island
CHD: congenital heart disease
CPC: Coding Potential Calculator
DEG: differentially expressed gene
DMR: differentially methylated region
DMW: differentially methylated window
DNase-Seq: DNase sequencing
FDR: false discovery rate
GO: Gene Ontology
HCM: hypertrophic cardiomyopathy
LA: left atrium
lncRNA: long noncoding RNA
LV: the left ventricle
MNase-seq: micrococcal nuclease sequencing
NDR: nucleosome-depleted region
NOMe-seq: nucleosome occupancy and methylome sequencing
PBAT: post-bisulfite adaptor tagging
PCA: principal component analysis
PVDF: polyvinylidene fluoride
qRT-PCR: quantitative reverse transcription PCR
RA: right atrium
RPKM: reads per kilobase per million
RRBS: reduced representation bisulfite sequencing
RV: right ventricle
TBS: Tris-buffered saline
TES: transcription end site
TF: transcription factor
TSS: transcription start site
WGBS: whole genome bisulfite sequencing
