# Supplementary figures and images for: Integrated transcriptomics and epigenomics reveal chamber-specific and species-specific characteristics of human and mouse hearts
Source: PLoS Biol. 2021 May 18;19(5):e3001229. doi: 10.1371/journal.pbio.3001229 (PMC8130971; doi:10.1371/journal.pbio.3001229)

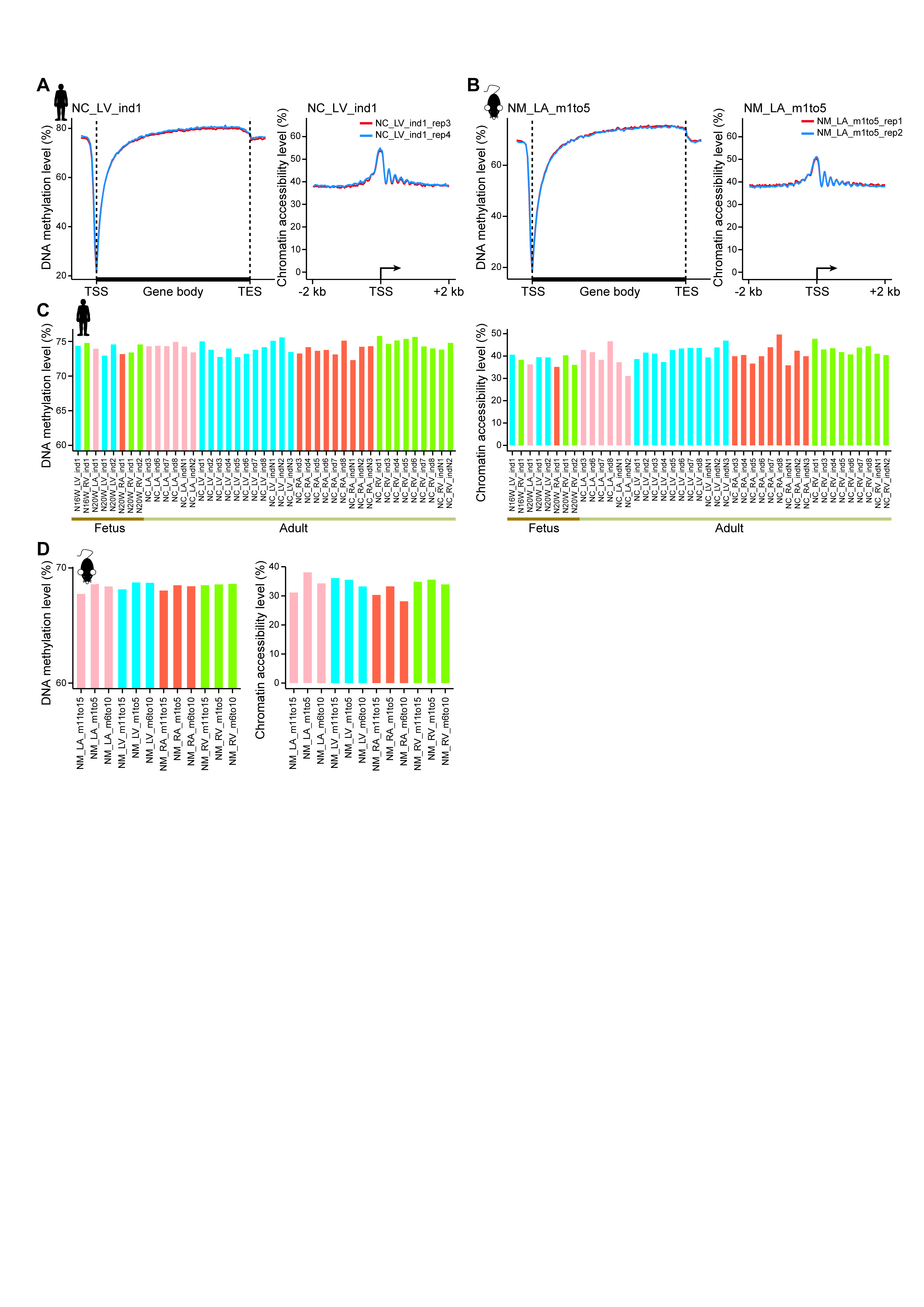

Supplement: S1 Fig — (A) Line plots showing the average endogenous DNA methylation level around the gene body ± 5 kb region (left) and the average chromatin accessibility levels around the TSS ± 2 kb region (right) in the adult human heart NC_LV_ind1. Different colors indicate different technology replications. (B) Line plots showing the average endogenous DNA methylation level around the gene body ± 5 kb region (left) and the average chromatin accessibility levels around the TSS ± 2 kb region (right) in the mouse heart NM_LA_m1to5. Different colors indicate different technology replications. (C) Bar plots showing the average endogenous DNA methylation levels (left) and the average chromatin accessibility levels (right) in the whole genome in the human heart. (D) Bar plots showing the average endogenous DNA methylation levels (left) and the average chromatin accessibility levels (right) in the whole genome in the mouse heart. The raw data for A–D can be found in S2 Data. LA, left atrium; LV, left ventricle; TES, transcription end site; TSS, transcription start site. (TIF) [file pbio.3001229.s001.tif]

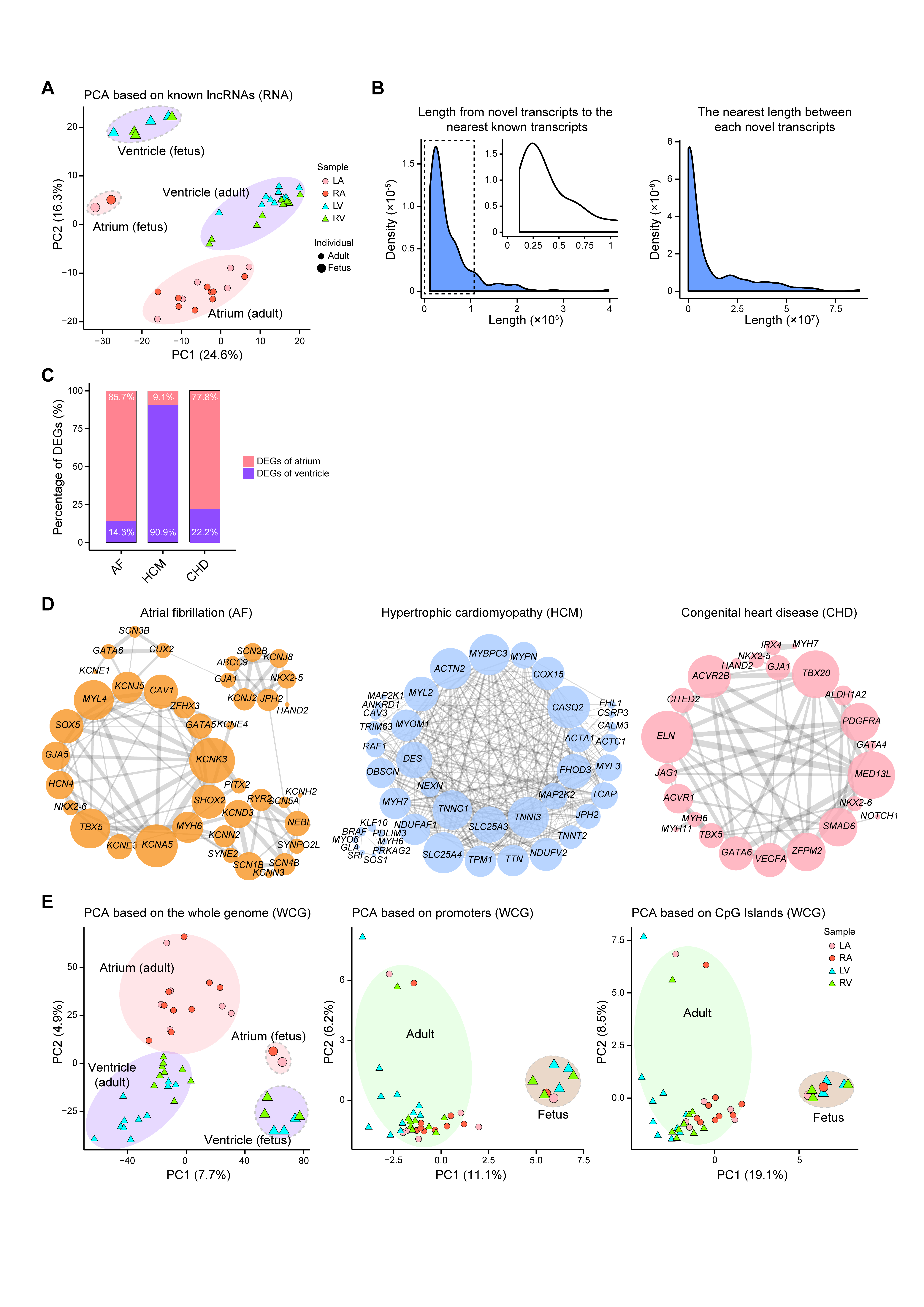

Supplement: S3 Fig — (A) PCA plot showing the transcriptome pattern of known lncRNAs in the human heart. Circles and triangles indicate atrium and ventricle samples, respectively; point sizes indicate the adult heart and fetal heart. The variation values of PC1 and PC2 were 24.6% and 16.3%, respectively. (B) Left, line plot showing the density distribution of length from novel transcripts to the nearest known transcripts; the zoom-out visualization of the dashed region is shown. Right, line plot showing the density distribution of the nearest length between each novel transcript. (C) Bar plot showing the distribution of human DEGs in heart disease-associated gene sets. These DEGs were identified with comparisons between the adult atria and ventricles. (D) Coexpression networks of cardiovascular diseases (AF, HCM, and CHD) associated genes. The point size indicated the number of connections for a given gene, and the line thickness indicated the correlation coefficient for a given gene pair. (E) PCA plot showing the endogenous DNA methylation pattern of the whole genome (left), promoters (middle), and CpG islands (right) in the human heart. Rounds and triangles indicate atrium and ventricle samples, respectively; point sizes indicate the adult heart and fetal heart. The raw data for A–E can be found in S2 Data. AF, atrial fibrillation; CHD, congenital heart disease; DEGs, differentially expressed genes; HCM, hypertrophic cardiomyopathy; LA, left atrium; lncRNAs, long noncoding RNAs; LV, left ventricle; PCA, principal component analysis; RA, right atrium; RV, right ventricle. (TIF) [file pbio.3001229.s003.tif]

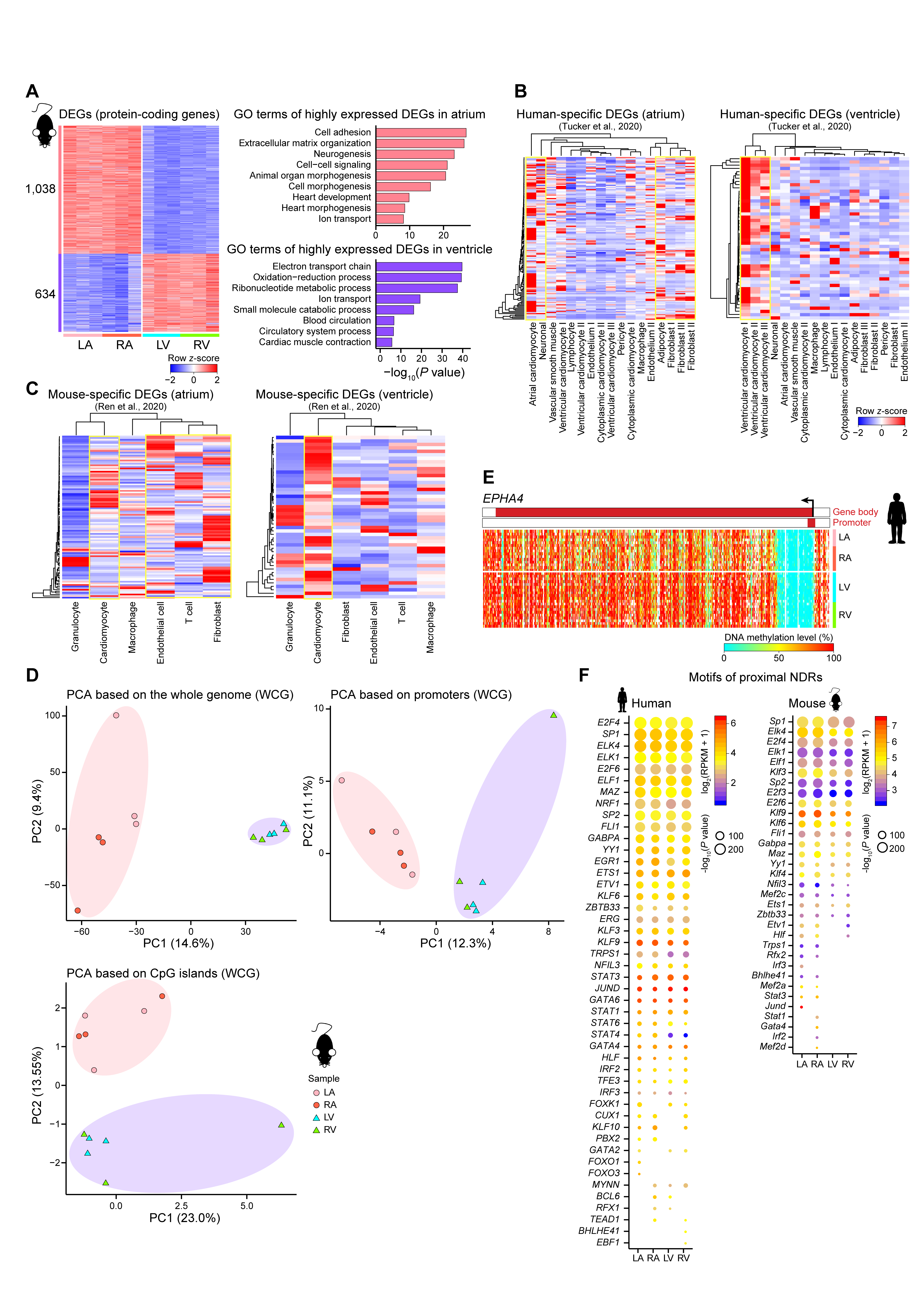

Supplement: S4 Fig — (A) Heatmap showing row z-score scaled gene expression levels of DEGs (protein-coding genes) between the atria and ventricles in the mouse heart (left), and corresponding GO terms are shown (right). The number of DEGs is reported on the left. (B) Heatmaps showing z-score scaled expression levels of human-specific DEGs among different cell types from single-cell RNA-seq data. Left: human-specific DEGs with higher expression levels in the atria; right, human-specific DEGs with higher expression levels in the ventricles. (C) Heatmaps showing z-score scaled expression levels of mouse-specific DEGs among different cell types from single-cell RNA-seq data. Left: mouse-specific DEGs with higher expression levels in the atria; right, mouse-specific DEGs with higher expression levels in the ventricles. (D) PCA plots showing the endogenous DNA methylation pattern of the whole genome, promoters, and CpG islands in the mouse heart. Circles and triangles indicate atrium and ventricle samples, respectively. (E) Heatmaps showing endogenous DNA methylation levels in the gene body ± 10 kb regions of EPHA4 in human hearts. The color bars in the heatmaps indicate the gene-body regions and the promoter regions (from 1 kb upstream of the TSS to 0.5 kb downstream of the TSS). (F) Motif enrichment analysis of proximal NDRs in the adult human heart (left) and mouse heart (right). Colors indicate average expression levels, and sizes indicate P values (P ≤ 10−10) of the corresponding transcription factor. The raw data for A–F can be found in S2 Data. DEGs, differentially expressed genes; GO, Gene Ontology; LA, left atrium; LV, left ventricle; NDRs, nucleosome-depleted regions; PCA, principal component analysis; RA, right atrium; RPKM, reads per kilobase per million; RV, right ventricle; TSS, transcription start site. (TIF) [file pbio.3001229.s004.tif]

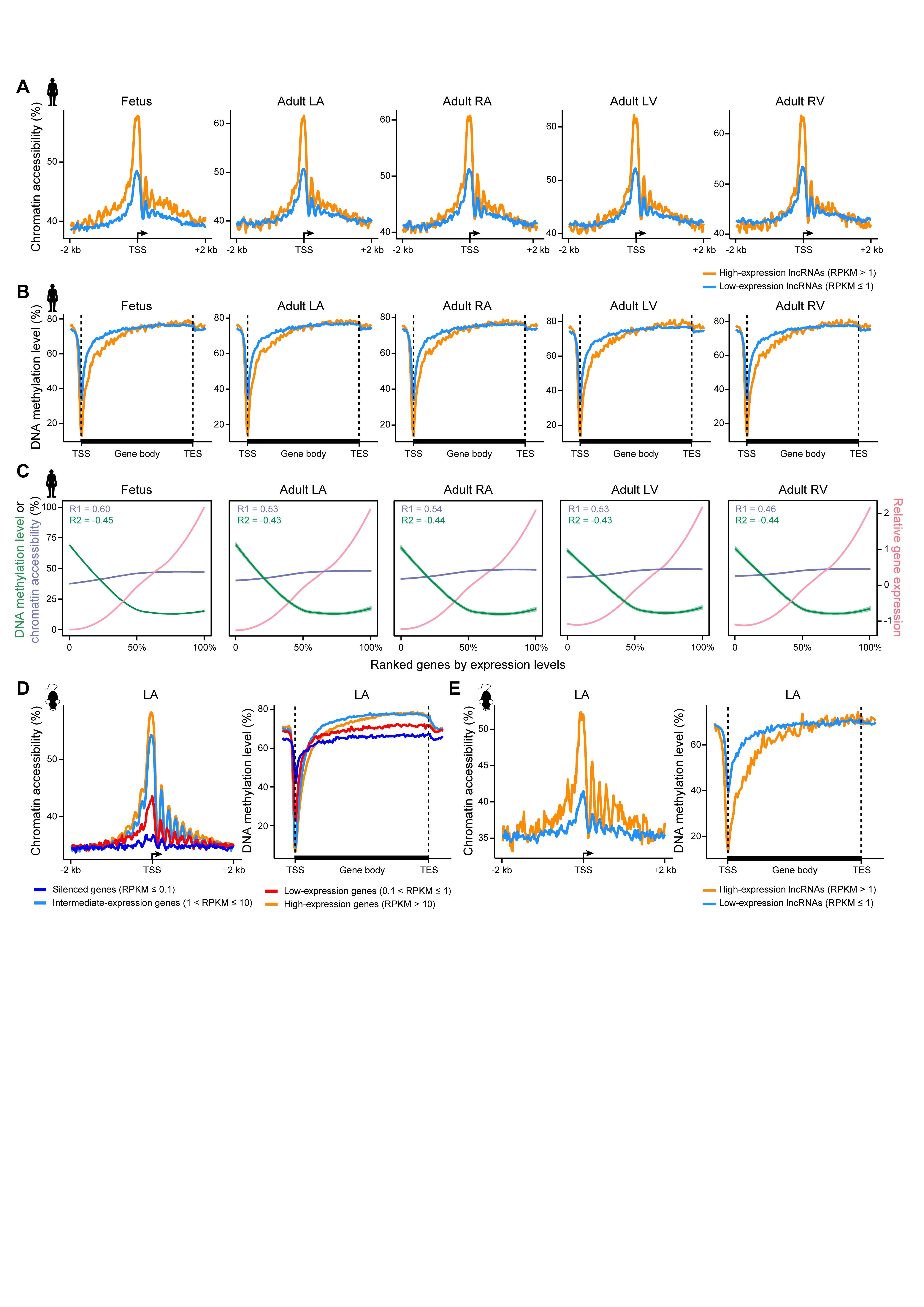

Supplement: S5 Fig — (A and B) Line plots showing the average chromatin accessibility level around the TSS ± 2 kb region (A) and the average endogenous DNA methylation level around the gene body ± 5 kb region (B) of lncRNAs in the human heart. Different colors indicate 2 lncRNA groups classified according to expression levels. (C) Line plots showing the average endogenous DNA methylation level (green) and the average chromatin accessibility level (blue) in promoters of corresponding genes. Genes were ranked by expression levels, and the x-axis from left to right represented genes with increased expression levels. Spearman correlation coefficients between chromatin accessibility levels and gene expression levels (R1) and Spearman correlation coefficients between endogenous DNA methylation levels and gene expression levels (R2) are indicated. (D) Line plots showing the average chromatin accessibility level around the TSS ± 2 kb region (left) and the average endogenous DNA methylation level around the gene body ± 5 kb region (right) of protein-coding genes in the mouse heart LA. Different colors indicate 4 gene groups classified according to expression levels. (E) Line plots showing the average chromatin accessibility level around the TSS ± 2 kb region (left) and the average endogenous DNA methylation level around the gene body ± 5 kb region (right) of lncRNAs in the mouse heart LA. Different colors indicate 2 lncRNA groups classified according to expression levels. The raw data for A–E can be found in S2 Data. LA, left atrium; lncRNAs, long noncoding RNAs; LV, left ventricle; RA, right atrium; RPKM, reads per kilobase per million; RV, right ventricle; TES, transcription end site; TSS, transcription start site. (TIF) [file pbio.3001229.s005.tif]
