# Supplementary material for: Integrated transcriptomics and epigenomics reveal chamber-specific and species-specific characteristics of human and mouse hearts
Source: PLoS Biol. 2021 May 18;19(5):e3001229. doi: 10.1371/journal.pbio.3001229 (PMC8130971; doi:10.1371/journal.pbio.3001229)
Supplement: S1 Raw images — (PDF) [file pbio.3001229.s015.pdf]

Full unedited Blot for Figure 4E

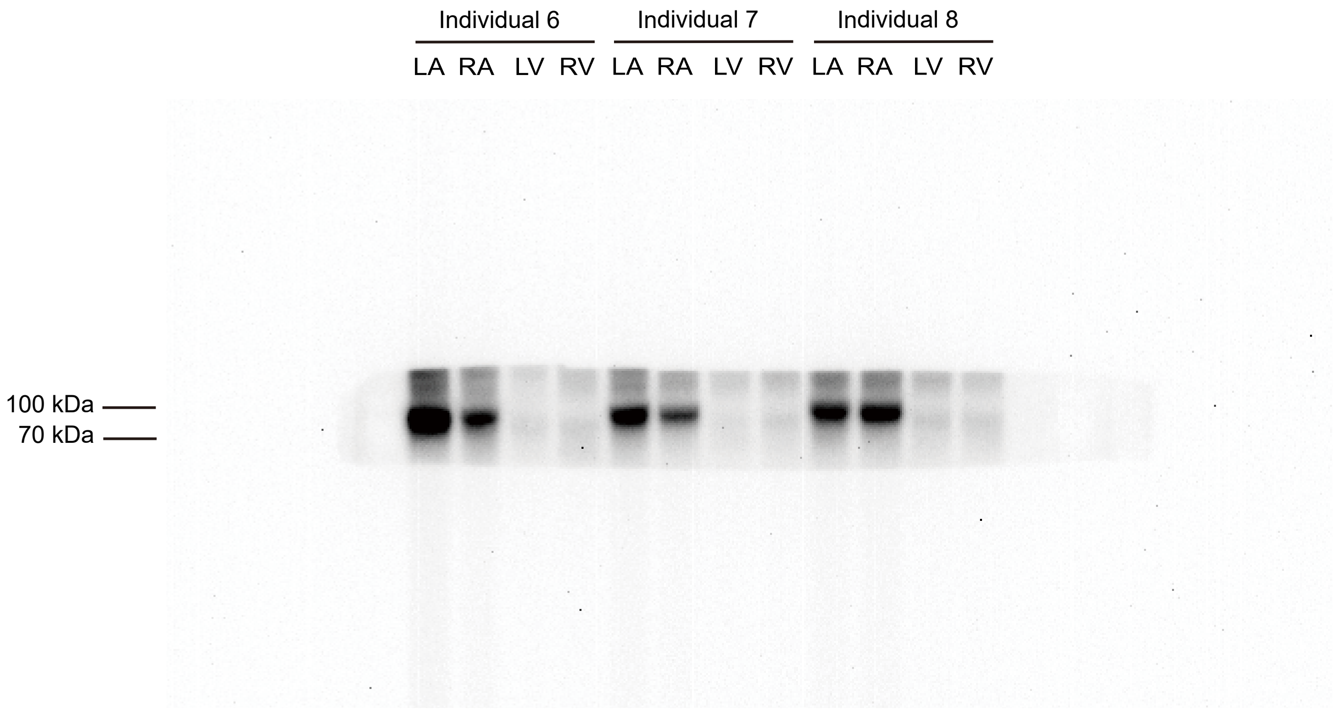

STAT4

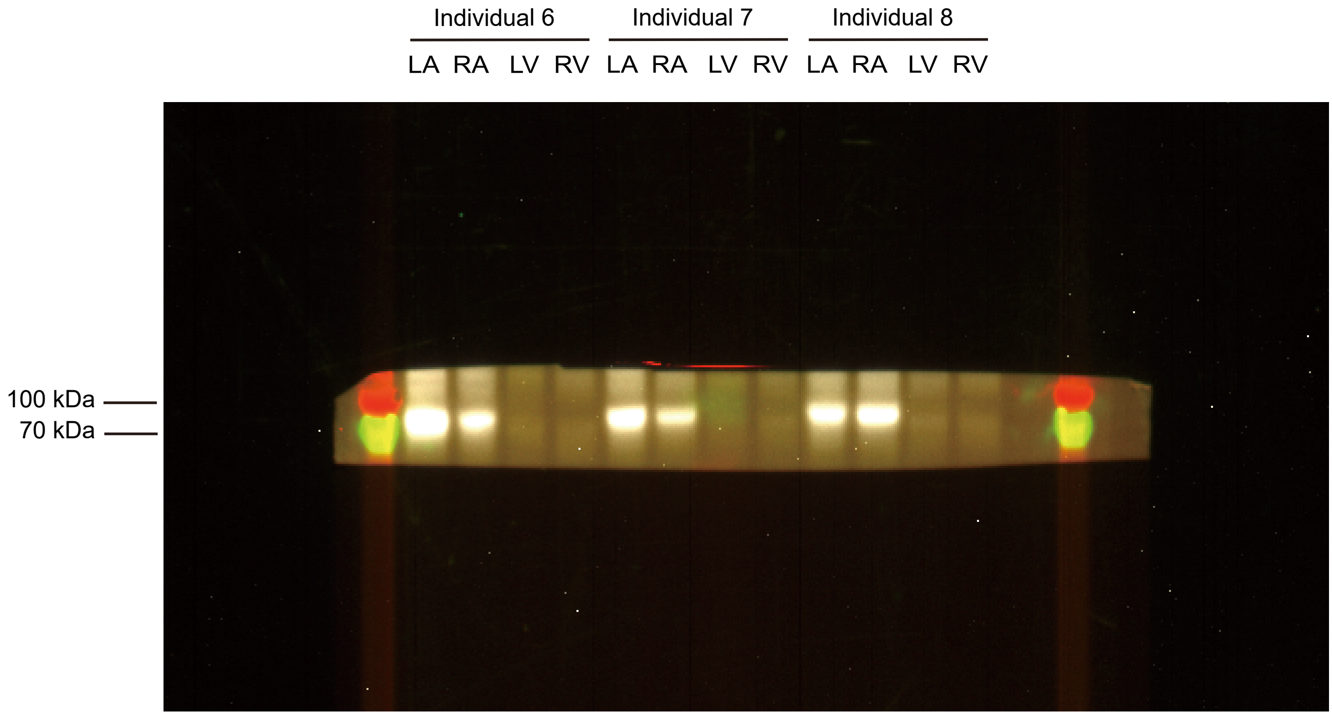

STAT4

Full unedited Blot for Figure 4E

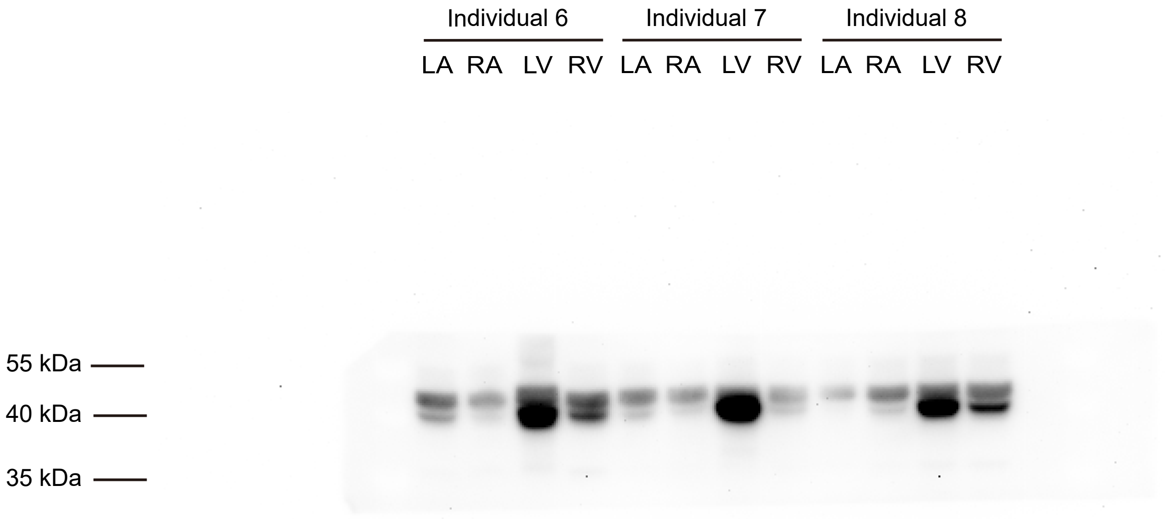

ANKRD2

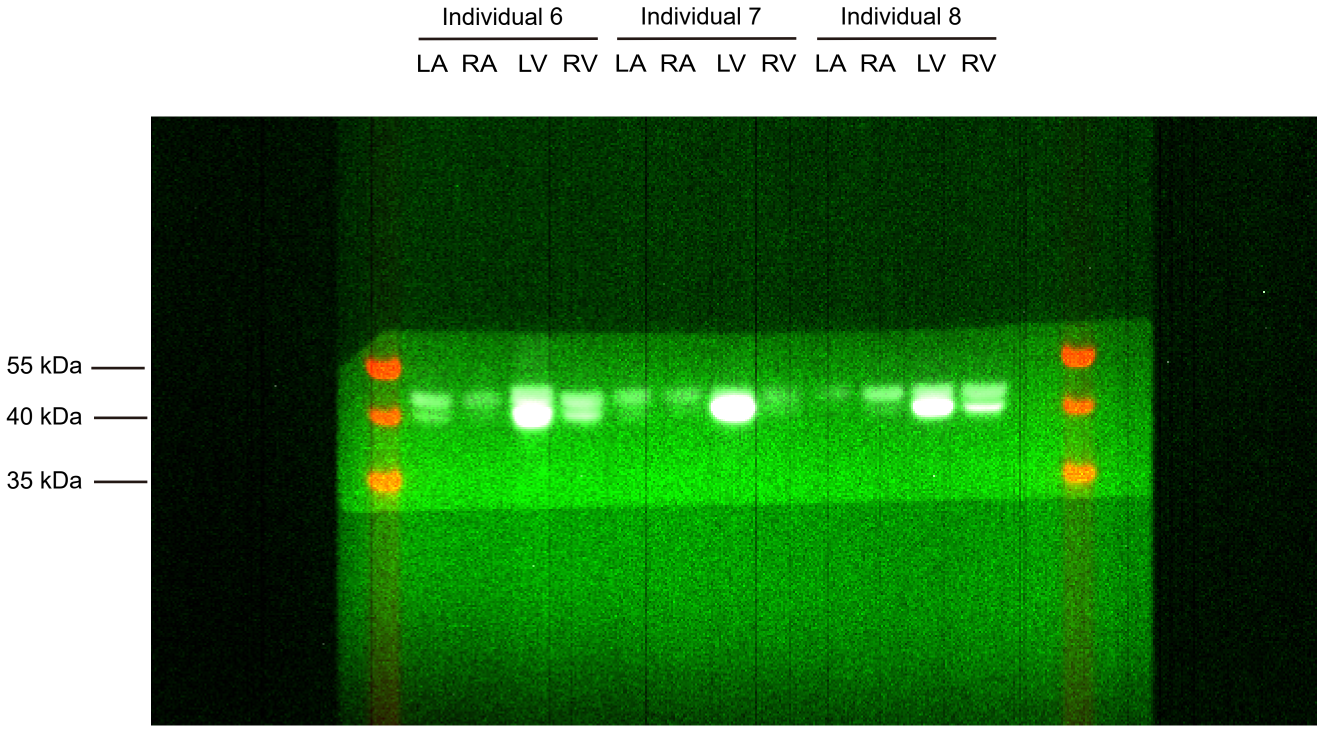

ANKRD2

Full unedited Blot for Figure 4E

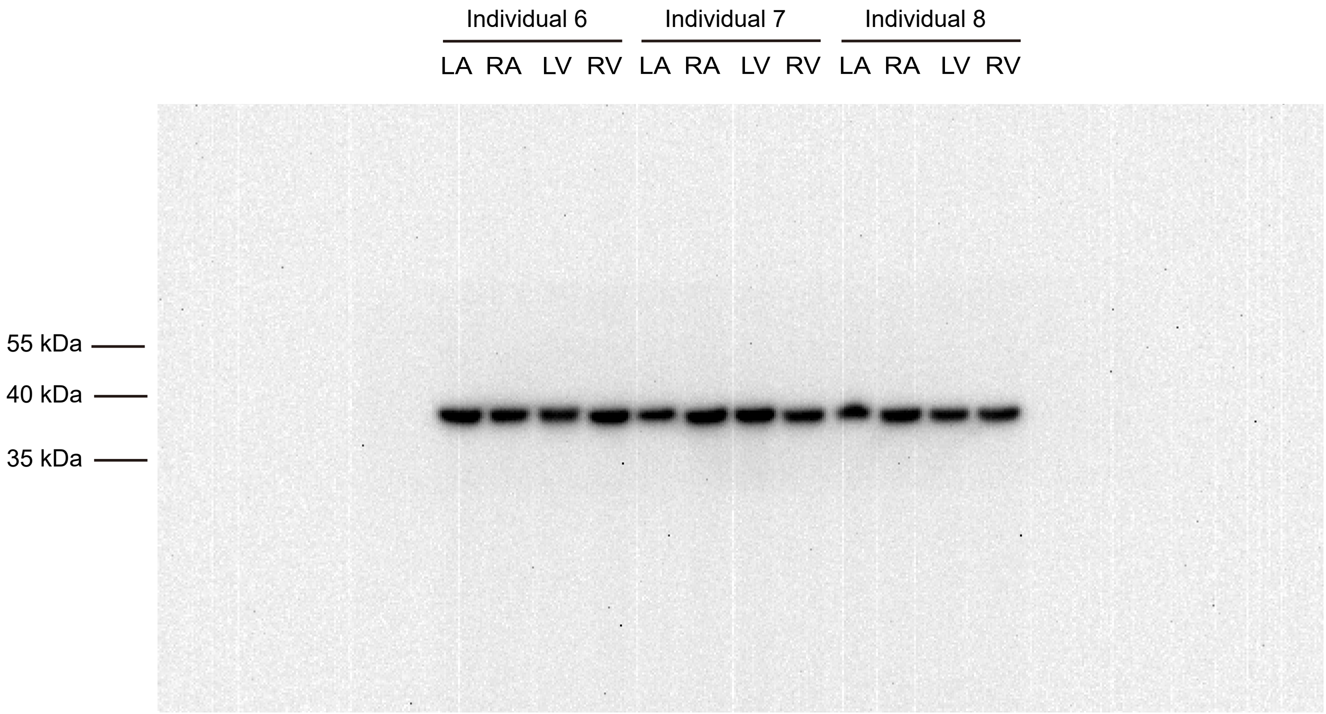

GAPDH

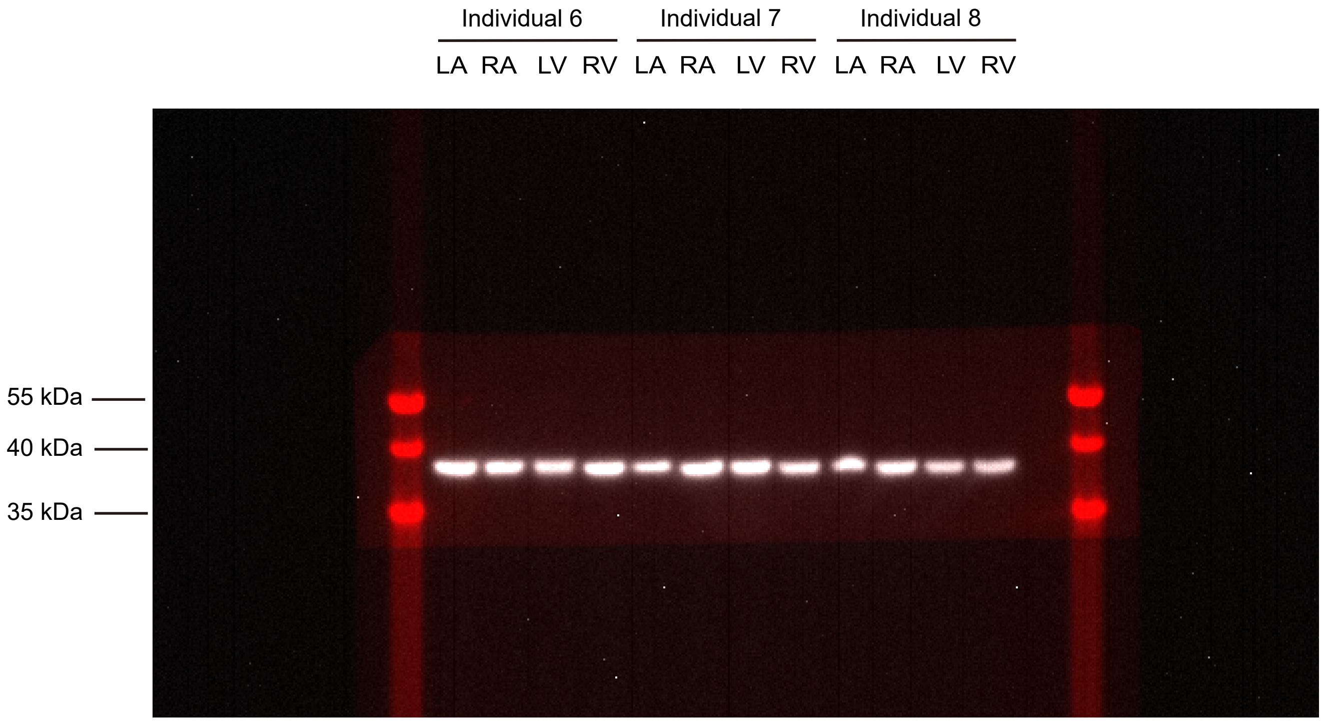

GAPDH
